# Long-Term Safety of Gantenerumab in Participants with Alzheimer’s Disease: A Phase III, Double-Blind, and Open-Label Extension Study (Marguerite RoAD)

**DOI:** 10.3233/JAD-240221

**Published:** 2024-08-27

**Authors:** Anuja Neve, Bibha Das, Jakub Wojtowicz, Zhiyue Huang, Szofia Bullain, Michelle Watkin, Dominik Lott, Tobias Bittner, Paul Delmar, Gregory Klein, Carsten Hofmann, Geoffrey A. Kerchner, Janice Smith, Monika Baudler, Paulo Fontoura, Rachelle S. Doody

**Affiliations:** a F. Hoffmann-La Roche Ltd, Basel, Switzerland; b F. Hoffmann-La Roche Ltd, Shanghai, China; c Roche Products Limited, Welwyn Garden City, UK; d Genentech, Inc., South San Francisco, CA, USA

**Keywords:** Alzheimer’s disease, clinical efficacy, clinical trial, gantenerumab, safety

## Abstract

**Background:**

Gantenerumab is an anti-amyloid-β immunoglobulin G1 monoclonal antibody for subcutaneous (SC) administration. The efficacy and safety of low-dose (105 mg or 225 mg) gantenerumab were investigated in Marguerite RoAD (MR; NCT02051608), a Phase III, double-blind (DB), placebo-controlled study in participants with mild Alzheimer’s disease (AD) dementia. Following a preplanned futility analysis of the SCarlet RoAD study (NCT01224106), MR was converted into an open-label extension (OLE).

**Objective:**

The DB study aimed to assess the efficacy of gantenerumab compared with placebo from baseline to Week 104 in participants with mild AD dementia. Following conversion to an OLE, this objective became exploratory, as the OLE assessed the long-term safety and tolerability of SC gantenerumab at doses of up to 1,200 mg every 4 weeks (Q4W) in OLE participants.

**Methods:**

Eligible DB study participants were offered the opportunity to receive gantenerumab up-titrated to 1,200 mg Q4W. Safety and tolerability were assessed using magnetic resonance imaging (MRI), physical and neurologic examinations, and adverse event monitoring.

**Results:**

Overall, 225 participants were rolled over from the DB part of MR and received ≥1 gantenerumab dose in the OLE. The median treatment duration was 123 weeks. Fifty-nine (26.2%) and 41 (18.2%) participants had amyloid-related imaging abnormality (ARIA)-edema and ARIA-hemorrhage MRI findings, respectively. ARIA findings were manageable with MRI monitoring and dose intervention; most were asymptomatic. There were no unexpected safety findings.

**Conclusions:**

SC gantenerumab at doses of up to 1,200 mg Q4W were well tolerated in participants with mild AD dementia.

## INTRODUCTION

Alzheimer’s disease (AD) is the most common type of dementia, accounting for 60–70% of all cases.^1^ It is estimated that 55 million people have dementia worldwide, with an expected increase to 139 million by 2050.^2^ AD and other forms of dementia are among the top 10 causes of death worldwide, ranking as the second leading cause of death in high-income countries.^3^ The clinical progression of AD follows a continuum from preclinical, without any overt symptoms, to mild cognitive impairment due to AD (also known as prodromal AD) then mild, moderate, and severe forms of AD dementia.^4^ The optimal time to treat people with AD is in the earlier stages of AD, to ensure cognitive and functional capabilities remain intact and to slow progression into more advanced stages.^5^

AD pathophysiology is characterized by a progressive accumulation of amyloid plaques, mainly comprising aggregated amyloid-β (Aβ) and neurofibrillary tangles, composed of aggregated pathologic tau protein, in the brain.^1,6^ There is growing evidence that elevated Aβ levels promote the accumulation of toxic forms of tau, eventually causing downstream neurodegeneration and dementia.^7^ Monoclonal antibodies that target different Aβ protein species in AD have been developed but have given mixed results in Phase II and Phase III clinical trials.^8–14^ Antibodies that result in a high proportion of individuals reaching amyloid negativity, assessed by amyloid positron emission tomography (PET), have shown benefit in slowing clinical and functional decline;^8–10,12,15^ however, drugs that do not fully remove amyloid plaques have shown little to no benefit.^11,13,14^

Gantenerumab is an investigational, fully human anti-Aβ immunoglobulin G1 monoclonal antibody for subcutaneous (SC) administration, with highest affinity for aggregated Aβ, including oligomers, fibrils, and plaques. It removes Aβ via microglia-mediated phagocytosis, and has shown downstream effects on biomarkers of AD pathology and neurodegeneration in clinical trials.^16–18^

The effects of SC low-dose (105 mg or 225 mg every 4 weeks [Q4W]) gantenerumab on cognition and function were investigated in two multicenter, randomized, double-blind, placebo-controlled studies in participants with early AD (SCarlet RoAD [SR] in participants with prodromal AD [NCT01224106]^19^ and Marguerite RoAD [MR] in participants with mild AD [NCT02051608]^20^). Dosing in the double-blind part of the SR study was halted prematurely on December 19, 2014, following a preplanned futility analysis.^6^

Data from the double-blind part of SR, together with external data from another anti-Aβ compound (aducanumab), suggested that the gantenerumab doses under study were subtherapeutic.^21^ Subgroup analyses indicated that higher exposure to gantenerumab may have clinically relevant effects on cognition and function in a subgroup of participants predicted to show fast progression as defined by a mathematic model developed by Delor et al. (2013).^6,22^

Recruitment in the double-blind part of MR was halted (October 2015), and both the MR and SR studies were converted into open-label extension (OLE) studies to evaluate the safety and tolerability of gantenerumab at higher SC doses of up to 1,200 mg Q4W for up to 5 additional years in participants receiving placebo or gantenerumab in the double-blind parts of the respective studies.

To support dose selection in the SR and MR OLEs, two exposure–response models were developed and several titration regimens were tested, with the aim of achieving significant reduction in amyloid PET signal after 1 year of treatment while ensuring an acceptable amyloid-related imaging abnormalities (ARIA)-edema (ARIA-E) event rate over 2 years for both apolipoprotein E *ɛ*4 allele (*APOE* *ɛ*4) carriers and noncarriers.^21,23^ The models used to inform dose selection and titration regimens for the MR OLE have been described previously.

Data from the SR and MR OLEs were used to inform the dose and titration scheme of the GRADUATE I and II (NCT03444870; NCT03443973) Phase III studies of gantenerumab.^24^ The results of the SR double-blind and OLE parts have been reported elsewhere^6^ (Boada M et al. unpublished data). Here, we report detailed safety and efficacy data along with the pharmacodynamic analyses from both the double-blind and OLE parts of the MR study that informed the higher doses used in subsequent studies.

## MATERIALS AND METHODS

### Study design and participants

MR (NCT02051608; registered January 01, 2014) was a Phase III, multicenter, randomized, double-blind, placebo-controlled, parallel-group study of gantenerumab in mild AD, which was followed by an OLE.

The participant screening period for the double-blind part lasted 8 weeks. In the double-blind part, participants were 50–90 years of age and had either a clinical diagnosis of probable mild AD (based on the National Institute of Neurological and Communicative Disorders and Stroke and the Alzheimer’s Disease and Related Disorders Association criteria), or a major neurocognitive disorder due to AD of mild severity (based on the Diagnostic and Statistical Manual of Mental Disorders [DSM-5] criteria), with evidence of amyloid pathology via cerebrospinal fluid Aβ_42_ assessment (see Supplementary Table 1 for inclusion and exclusion criteria).

**Fig. 1:**
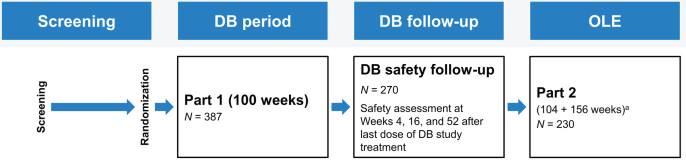
Overview of study design for the double-blind and OLE stages. ^a^In addition to the initial 2 years in OLE, participants were given the option to continue receiving open-label gantenerumab treatment until the end of 2020. Participants who discontinued study drug at any time during OLE or who completed the first 2 years of OLE only were asked to complete follow-up visits at 4 and 16 weeks from their last dose. Participants who received open-label gantenerumab for an additional 3 years beyond the initial 2 years of OLE were given the option of enrolling in an open-label rollover study (Open RoAD [NCT04339413]) aimed at evaluating the safety and tolerability of long-term administration of gantenerumab. Participants who rolled over to Open RoAD had one follow-up visit 4 weeks following the last dose, and those who did not had follow-up visits at 4 and 16 weeks from their last dose. DB, double-blind; OLE, open-label extension.

In the double-blind part of the study, participants were randomized to receive SC gantenerumab or placebo Q4W for 100 weeks, with a 52-week follow-up period after the final dose (Fig. 1). Following the preplanned interim futility analysis for the SR study, SR was converted to an OLE. Based on this analysis, enrollment in the double-blind part of MR was suspended while dosing continued; the study was also converted to an OLE. Participants who had already been randomized and were actively participating in the double-blind part of the study were offered the opportunity to participate in the OLE. Participants were not eligible for the OLE if they had discontinued from the double-blind part of the study (Supplementary Table 1). In contrast to the participants from SR, participants continuing from the double-blind part of MR into the OLE did not have an off-study period.

The study was approved by individual institutional ethics committees or institutional review boards and was conducted in accordance with the principles of the Declaration of Helsinki and good clinical practice.

### Titration regimen and dose administration

In the double-blind part of the study, participants in the active arm received SC gantenerumab at a dose of 105 mg Q4W SC for the first 24 weeks. Following magnetic resonance imaging (MRI) after the Week 24 dose, if no ARIA-E and no more than one new microhemorrhage were detected, then the participant was eligible for dose up-titration to 225 mg Q4W SC for the remainder of the study, regardless of the *APOE* *ɛ*4 status. However, given the decision to convert the double-blind part of the study to an OLE, not all participants could be up-titrated to 225 mg and, thus, remained at 105 mg at the time of rollover to the OLE. Participants who were not eligible for dose titration continued to receive 105 mg Q4W from Week 28 until the end of thestudy.

**Fig. 2:**
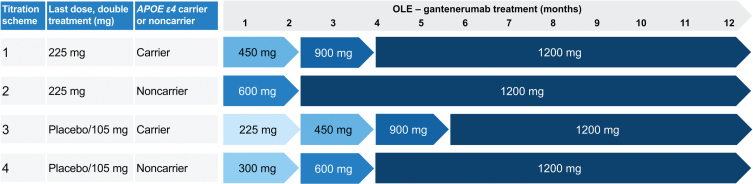
Up-titration regimens for Marguerite RoAD OLE. MRI performed prior to each dose increase and then at regular intervals. Implementation of dosing algorithms in case of ARIA findings. ARIA, amyloid-related imaging abnormalities; *APOE* *ɛ*4, apolipoprotein E *ɛ*4 allele; MRI, magnetic resonance imaging; OLE, open-label extension. Adapted from Bateman RJ, et al. Alz Res Therapy 14, 178 (2022) under the terms of the Creative Commons CC BY license https://creativecommons.org/licenses/by/4.0/.

In the OLE, participants were up-titrated to doses of up to 1,200 mg Q4W SC (Fig. 2). Four up-titration regimens were included in the OLE, based on participants’ treatment assignments (gantenerumab or placebo), last dose received in the double-blind part of the study, and *APOE* *ɛ*4 status (noncarriers [0*ɛ*4] and carriers of one or two alleles [1*ɛ*4 or 2*ɛ*4]) (Fig. 2).

Doses of 105 and 225 mg were administered SC by a prefilled syringe or from a vial.^25^ The 450 mg and 900 mg doses could be administered SC by prefilled syringe or from vials using an infusion pump. The 1,200 mg gantenerumab dose was administered SC from vials using an infusion pump.

### Outcome measures

The primary objective in the double-blind part was to evaluate the efficacy of gantenerumab compared with placebo from baseline to Week 104. For details on the outcome measures in the double-blind part, please refer to the Supplementary Material.

The primary objective of the OLE was to evaluate the safety and tolerability of gantenerumab at higher doses, up to 1,200 mg Q4W. Safety and tolerability were assessed by MRI, physical and neurologic examinations, vital signs, blood safety tests, electrocardiograms, Columbia Suicide Severity Rating Scale, and adverse event (AE) reporting. AEs were coded using the Medical Dictionary for Regulatory Activities version 24.0.

Secondary outcomes included the effect of 1,200 mg gantenerumab SC Q4W over time on brain amyloid load by PET imaging and on clinical outcomes (cognition and function) compared with the start of the OLE; pharmacokinetics (PK); and the incidence of anti-drug antibodies with higher gantenerumab doses in the target participant population. A full schedule of assessments is provided in Supplementary Table 2.

### MRI monitoring and management of MRI findings

All MRI scans (in the double-blind part of MR and the OLE) were read by a central reader, and information about potential central nervous system (CNS) symptoms was collected prospectively (i.e., the site contacted the participant up to 1 week prior to each MRI).

Prespecified dose intervention rules were included in the double-blind and OLE parts of the study for both ARIA-E and ARIA-hemorrhage (ARIA-H; Table 1). Please refer to the Supplementary Material for MRI monitoring and management of ARIA findings in the double-blind part of the study.

**Table 1:** Rules for ARIA management in the MR double-blind part and OLE

Double-blind part		
Event	Characteristics	Action
ARIA-E	Single ARIA-E ≤2 cm	Continue study drug at the same dose level. Perform monthly monitoring until ARIA-E has resolved
	ARIA-E >2 cm, or multiple ARIA-E	Withhold study drug. If ARIA-E has resolved or significantly decreased and stabilized, restart dosing at 105 mg Q4W. If ARIA-E recurs, discontinue treatment
ARIA-H	Two new microhemorrhages in a single scan or 3–4 microhemorrhages cumulatively	For participants on 225 mg gantenerumab, reduce dose to 105 mg
	>5 microhemorrhages cumulatively	Discontinue from study drug
OLE		
Event	Characteristics	Action
ARIA-E	Asymptomatic^a^ ARIA-E with size >1 and <4 BGTS	Continue study drug at the same dose level (i.e., do not up-titrate)
		Repeat MRI 4 weeks later
		If ARIA-E is stable or decreased, continue study drug at the same dose level, and continue monthly MRI monitoring until event resolves^b^
		If ARIA-E resolves, resume up-titration as per SoA
		If ARIA-E increases (≥4 BGTS) or symptoms develop, refer to the rules below
	Symptomatic^a^ ARIA-E (any size), or asymptomatic ARIA-E with size ≥4 BGTS	Temporarily interrupt study drug and implement monthly MRI monitoring until event resolves
		Once symptoms and ARIA-E resolve, reintroduce study drug at same dose as that given at the time the event was detected, and perform an MRI after 1 month of dosing. If no new ARIA-E is detected, resume up-titration and MRI monitoring as per SoA
	Any new onset of ARIA-E	Treat the same as the first event (based on symptoms and BGTS)
ARIA-H	>8 microhemorrhages cumulatively^c^	Suspend titration (i.e., no further up-titration) for subjects on 450 mg gantenerumab or higher
		Permanently discontinue study treatment for subjects on 105 mg or 225 mg gantenerumab
	>10 microhemorrhages cumulatively^c^	Discontinue from study drug

In case both ARIA-E and ARIA-H occurred, the more conservative management approach was used. The investigators could choose to perform additional MRI monitoring for ARIA at any time; each titration step was preceded by a safety MRI. ^a^As per investigator judgment, CNS symptoms collected prospectively. ^b^ARIA-E resolved (BGTS of 0 or 1). ^c^Sum of ARIA-H at OLE baseline and newly detected ARIA-H post-OLE baseline during the study. ARIA-E, amyloid-related imaging abnormalities-edema; ARIA-H, amyloid-related imaging abnormalities-hemorrhage; BGTS, Barkhof Grand Total Scale; CNS, central nervous system; MR, Marguerite RoAD; MRI, magnetic resonance imaging; OLE, open-label extension; Q4W, every 4 weeks; SoA, schedule of assessments.

In the OLE, in the case of asymptomatic ARIA-E with a Barkhof Grand Total Scale score of <4,^26^ dosing could continue at the same dose level, but no up-titration could occur. If the ARIA-E was symptomatic or with a Barkhof Grand Total Scale score of ≥4, dosing was temporarily interrupted; reintroduction of dosing/titration occurred once ARIA-E was considered resolved. For ARIA-H findings, if the cumulative number of ARIA-H reached more than eight at dose levels below 450 mg, study treatment was discontinued; at dose levels of 450 mg or higher, further up-titration was suspended. If the cumulative number of ARIA-H reached more than 10, study treatment was discontinued.

Not all ARIA-E, ARIA-H, or other MRI findings were reportable AEs. These were only reportable as AEs if they met one or more of the following criteria: were accompanied by clinical symptoms; led to a change in study treatment (e.g., dose interruption or permanent discontinuation); required a change in concomitant therapy; resulted in a medical intervention; or were otherwise clinically significant in the investigator’s judgment.

For ARIA-E analysis, ARIA-E events reported as AEs were mapped to the following terms: “amyloid-related imaging abnormality-edema/effusion”; or “vasogenic cerebral edema”. For ARIA-H analysis, ARIA-H events were mapped to the following terms: “amyloid-related imaging abnormality-microhemorrhages and hemosiderin deposits”; “cerebellar microhemorrhage”; “cerebral microhemorrhage”; or “cerebral hemosiderin deposition”.

### Imaging assessments

Changes in brain amyloid load over time were assessed using florbetapir ^18^F (AV-45; Amyvidtrademark) and compared with baseline. In the double-blind part of the study, a subset of eligible participants enrolled in the study also participated in a PET substudy and underwent PET imaging scans four times during the substudy (at baseline, Week 52, Week 104, and Week 152). All participants entering OLE were invited to take part in the PET substudy, independent of their previous participation during the double-blind treatment period. This objective was evaluated annually during the initial 3 years ofthe OLE.

MRI was used in both the double-blind part and the OLE to assess the effects of gantenerumab over time on hippocampal volume, whole brain volume, ventricular volume, and other volumetric measures of the brain compared with the double-blind part screening.

### Cognitive and functional assessments

Please refer to the Supplementary Material for details of cognitive and functional assessments in the double-blind part of the study.

In the OLE, cognition was assessed using the Alzheimer Disease Assessment Scale – Cognitive Subscale 13, Clinical Dementia Rating, Alzheimer’s Disease Cooperative Study – Activities of Daily Living Scale during the initial 3 years, and Mini-Mental State Exam for the entire duration of the OLE.

### PK assessments

In both the double-blind and OLE parts of the study, plasma samples were analyzed for target binding competent gantenerumab concentrations using validated enzyme-linked immunosorbent assay methods. The development of anti-drug antibodies was assessed, and the impacts of these on PK and safety were explored.

### Statistical analysis

Please refer to the Supplementary Material for statistical methods for the double-blind part of the study.

For the OLE, the analysis population was the safety-evaluable (SE) population, which comprised participants who received at least one dose of gantenerumab during the OLE. There was no formal hypothesis testing in the OLE portion of the study. The analyses of ARIA events were summarized by the up-titration scheme assigned in the OLE or *APOE* *ɛ*4 status to inform the incidence of ARIA findings and provide further supporting evidence to the ARIA management guidance. Non-ARIA analyses were summarized by cohort. These reflected the treatment assignment that participants received during the double-blind part of the study, as neither the up-titration scheme nor *APOE* *ɛ*4 status was considered to bear a significant impact on the non-ARIA analyses, which focused on change in outcomesover time.

## RESULTS

This section will summarize the key results only from the OLE. For the double-blind part of the study, please refer to the Supplementary Material.

### Study population

**Fig. 3:**
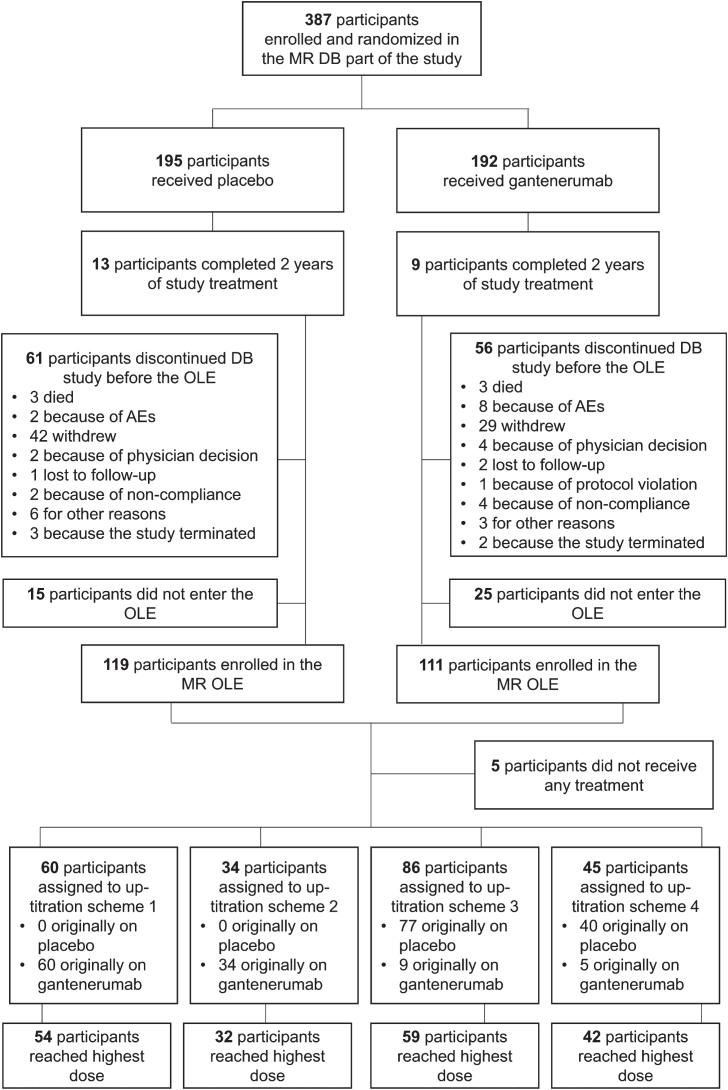
Disposition of participants in the MR DB part and OLE. AE, adverse event; DB, double-blind; MR, Marguerite RoAD; OLE, open-label extension.

Of the 387 participants who were randomized and received treatment in the double-blind phase, 230 (59.4%) participated in the OLE (Fig. 3). Of the 230 participants enrolled in the OLE, 225 received at least one dose of gantenerumab and were thus included in the SE analysis population. A total of 219 participants in the SE population also had at least one post-OLE baseline MRI.

### Baseline characteristics

Of the 230 participants in the OLE, 52% had received placebo and 48% had received gantenerumab (last dose: 105 mg [13%] or 225 mg [87%]) in the double-blind of the study (Table 2)).

**Table 2:** Demographic characteristics by previous randomization at OLE baseline

Characteristic	Participants previously on placebo (*n* = 117)	Participants previously on gantenerumab (*n* = 108)	Total (*N* = 225)
Treatment in DB part of MR
Placebo, *n*	117	0	117
Gantenerumab 105 mg, *n*	0	14	14
Gantenerumab 225 mg, *n*	0	94	94
Age, y, mean (SD)	71.8 (8.1)	71.0 (9.3)	71.4 (8.7)
Male, *n* (%)	48 (41.0)	47 (43.5)	95 (42.2)
Female, *n* (%)	69 (59.0)	61 (56.5)	130 (57.8)
Race, *n* (%)
White	94 (80.3)	85 (78.7)	179 (79.6)
Asian	19 (16.2)	19 (17.6)	38 (16.9)
Black	2 (1.7)	2 (1.9)	4 (1.8)
Pacific Islander	0	1 (0.9)	1 (0.4)
Other	2 (1.7)	1 (0.9)	3 (1.3)
Education level below undergraduate degree, *n* (%)
>12 y	70 (60.0)	58 (53.7)	128 (56.9)
*APOE* genotype, *n* (%)
0*ɛ*4	40 (34.2)	42 (38.9)	82 (36.4)
1*ɛ*4	58 (49.6)	45 (41.7)	103 (45.8)
2*ɛ*4	19 (16.2)	21 (19.4)	40 (17.8)
ARIA-E during DB, *n* (%)	115 (98.3)	94 (87.0)	209 (92.9)
Cumulative ARIA-H at OLE baseline, *n* (%)
0	95 (81.2)	84 (77.8)	179 (79.6)
1	12 (10.3)	14 (13.0)	26 (11.6)
2	3 (2.6)	4 (3.7)	7 (3.1)
3	4 (3.4)	4 (3.7)	8 (3.6)
≥4	3 (2.6)	2 (1.8)	5 (2.2)
Duration in DB part, weeks, mean (SD)	71.7 (22.6)	71.8 (20.3)	71.8 (21.5)
Time from last dose in DB part to first dose in OLE, weeks, mean (SD)	10.3 (12.4)	7.2 (8.2)	8.8 (10.7)
CDR-SB, mean (SD)	5.86 (2.87)	5.66 (2.68)	5.77 (2.77)
CDR-GS, mean (SD)	0.98 (0.53)	0.97 (0.47)	0.98 (0.50)
MMSE, mean (SD)	19.78 (5.21)	19.56 (4.79)	19.67 (5.00)
ADAS-Cog13, mean (SD)	35.17 (11.39)	36.13 (10.81)	35.63 (11.10)

ADAS-Cog13, Alzheimer’s Disease Assessment Scale – Cognitive Subscale 13; *APOE*, apolipoprotein E allele; ARIA-E, amyloid related imaging abnormalities-edema; ARIA-H, amyloid related imaging abnormalities-hemorrhage; CDR-GS, Clinical Dementia Rating – Global Score; CDR-SB, Clinical Dementia Rating – Sum of Boxes; DB, double-blind; MMSE, Mini-Mental State Examination; MR, Marguerite RoAD; OLE, open-label extension; SD, standard deviation.

Participants had a median age at OLE baseline of 72 years; the majority were female (57.8%) and White (79.6%). The *APOE* 1*ɛ*4 genotype was the most common (45.8%), followed by 0*ɛ*4 (36.4%) and 2*ɛ*4 (17.8%). Most participants (92.9%) had not experienced an ARIA-E during the double-blind part of the study, and 79.6% did not have ARIA-H at the time of rollover into the OLE.

The use of any approved symptomatic treatments for AD was permitted during the OLE. Please see the Supplementary Material for further details of the symptomatic treatments initiated during the OLE.

### Summary of AEs

In the OLE, the median duration of exposure to study treatment was 2.36 years (123 weeks) and the median number of treatment administrations was 30. Of the 225 participants who received gantenerumab in the OLE, 210 participants (93.3%) reported at least one AE.

The most frequently reported treatment-related AEs were injection site reactions (80 [35.6%] participants); ARIA-E (59 [26.2%] participants), and ARIA-H (41 [18.2%] participants). Fifty-four (24.0%) participants experienced AEs leading to dose intervention, with ARIA-E and ARIA-H being the most frequent (49 [21.8%] and 11 [4.9%] participants, respectively). Thirty-one (13.8%) participants were withdrawn from treatment following an AE, with the most frequent AEs being ARIA-H (12 [5.3%] participants) and ARIA-E (10 [4.4%] participants) (Table 3). Discontinuation of study treatment was mandated by the protocol when the cumulative number of new MRI findings of ARIA-H exceeded protocol-specified thresholds (see Methods). There were no mandatory ARIA-E–related discontinuation rules.

**Table 3:** Overview of AEs in the OLE by previous randomization (safety-evaluable population)

	Participants previously on placebo (*n* = 117)	Participants on gantenerumab (*n* = 108)	Total (*N* = 225)
Administration-related reaction	48 (41.0)	44 (40.7)	92 (41.0)
Total number of participants with at least one AE	107 (91.5)	103 (95.4)	210 (93.3)
Any AE, *n*	990	1038	2028
Deaths	5 (4.3)	5 (4.6)	10 (4.4)
Participants withdrawn from study due to an AE	14 (12.0)	17 (15.7)	31 (13.8)
Any serious AE	29 (24.8)	41 (38.0)	70 (31.1)
Any serious AE leading to withdrawal from treatment	4 (3.4)	6 (5.6)	10 (4.4)
Any serious AE leading to dose modification/interruption	7 (6.0)	8 (7.4)	15 (6.7)
Any related serious AE	4 (3.4)	7 (6.5)	11 (4.9)
Any AE leading to withdrawal from treatment	14 (12.0)	17 (15.7)	31 (13.8)
Any AE leading to dose modification/interruption	32 (27.4)	38 (35.2)	70 (31.1)
Any related AE	68 (58.1)	68 (63.0)	136 (60.4)
Any related AE leading to withdrawal from treatment	10 (8.5)	14 (13.0)	24 (10.7)
Any related AE leading to dose modification/interruption	23 (19.7)	30 (27.8)	53 (23.6)
Any severe AE	22 (18.8)	31 (28.7)	53 (23.6)
Participants with at least one of the following:
ARIA-H AE	19 (16.2)	24 (22.2)	43 (19.1)
ARIA-E AE	31 (26.5)	32 (29.6)	63 (28.0)

AE, adverse event; ARIA-E, amyloid-related imaging abnormalities-edema; ARIA-H, amyloid-related imaging abnormalities-hemorrhage; OLE, open-label extension.

Seventy participants experienced serious AEs (31.1%), of which 11 participants had 13 serious AEs that were assessed by the principal investigator as related to study treatment. Four serious symptomatic ARIA cases were reported and all resulted in hospitalization. There were 10 deaths (4.4%; due to myocardial infarction [in two participants], acute cardiac failure, cerebellar tumor, colon cancer, myelodysplastic syndrome, dementia Alzheimer’s type, dysphagia, subdural hematoma, and aortic aneurysm rupture); all were considered unrelated to study treatment by the principal investigator. No clinically relevant changes from OLE baseline were observed for any of the laboratory parameters and electrocardiograms (data on file). For the most frequently reported AEs (≥5% of participants), please refer to Supplementary Table 3.

### Incidence of ARIA MRI findings

Of the 225 participants in the OLE, 219 had at least one post-baseline MRI scan. Not all ARIA-E or ARIA-H cases were reportable as AEs (see Methods); hence, data in this section are based on ARIA-E and ARIA-H MRI findings. Overall, 70 (31.9%) participants had a new post-baseline ARIA-E MRI finding in the OLE, and 75 (34.2%) participants had a new post-baseline ARIA-H MRI finding in the OLE. Forty-eight (21.9%) participants had both new post-baseline ARIA-E and ARIA-H MRI findings in the OLE, not necessarily concurrently (Table 4).^27^

**Table 4:** ARIA findings by *APOE* *ɛ*4 carrier status and up-titration scheme in the OLE^a^

	Up-titration 1	Up-titration 2	Up-titration 3	Up-titration 4	Total
Median (range) ARIA-E radiologic severity (assessed by BGTS)^b^	5.00 (1.0–40.0)	10.00 (1.0–25.0)	15.50 (1.0–32.0)	9.50 (4.0–20.0)	9.00 (1.0–40.0)
New ARIA-E event, *n*/*N* (%)
Carriers^c^	22/58 (37.9)	–	29/80 (36.3)	–	51/138 (37.0)
1*ɛ*4	11/38 (28.9)	–	17/62 (27.4)	–	28/100 (28.0)
2*ɛ*4	11/20 (55.0)	–	12/18 (66.7)	–	23/38 (60.5)
Noncarriers	–	9/33 (27.2)	0/3 (0)	10/45 (22.2)	19/81 (23.5)
Recurrent ARIA-E event, *n*/*N* (%)
Carriers^c^	5/58 (8.6)	–	8/80 (10.0)	–	13/138 (9.4)
1*ɛ*4	3/38 (7.9)	–	5/62 (8.1)	–	8/100 (8.0)
2*ɛ*4	2/20 (10.0)	–	3/18 (16.7)	–	5/38 (13.2)
Noncarriers	–	1/33 (3.0)	0/3 (0)	1/45 (2.2)	2/81 (2.5)
New ARIA-H event, *n*/*N* (%)
Carriers^c^	23/58 (39.6)	–	37/80 (46.3)	–	60/138 (43.5)
1*ɛ*4	13/38 (34.2)	–	27/62 (43.5)	–	40/100 (40.0)
2*ɛ*4	10/20 (50.0)	–	10/18 (55.6)	–	20/38 (52.6)
Noncarriers	–	8/33 (24.2)	1/3 (33.3)	6/45 (13.3)	15/81 (18.5)
New ARIA-E and ARIA-H event, *n*/*N* (%)
Carriers^c^	17/58 (29.3)	–	24/80 (30.0)	–	41/138 (29.7)
1*ɛ*4	9/38 (23.7)	–	15/62 (24.2)	–	24/100 (24.0)
2*ɛ*4	8/20 (40.0)	–	9/18 (50.0)	–	17/38 (44.7)
Noncarriers	–	4/33 (12.1)	0/3 (0)	3/45 (6.7)	7/81 (8.6)

^a^Refer to Fig. 2 for titration schemes. ^b^BGTS radiologic severity scale correlates to three-point severity scale used in other studies.^27^ ^c^Includes those with either one (1*ɛ*4) or two (2*ɛ*4) copies of the *APOE* *ɛ*4. *APOE* *ɛ*4, apolipoprotein E *ɛ*4 allele; ARIA, amyloid-related imaging abnormalities; ARIA-E, amyloid-related imaging abnormalities – edema; ARIA-H, amyloid-related imaging abnormalities – hemorrhage; BGTS, Barkhof Grand Total Scale; OLE, open-label extension.

Most ARIA-E findings were seen at higher doses and in *APOE* *ɛ*4 carriers (Table 4 and Supplementary Table 4). Of the 138 *APOE* *ɛ*4 carriers, 51 (36.9%; 22 participants in up-titration scheme 1 and 29 in up-titration scheme 3) had ARIA-E MRI findings and 60 (43.4%; 23 participants in up-titration scheme 1 and 37 in up-titration scheme 3) participants had ARIA-H MRI findings. *APOE* *ɛ*4 homozygotes demonstrated higher incidences of both ARIA-E and ARIA-H compared with heterozygotes (Table 4). Of the 81 *APOE* *ɛ*4 noncarriers, 19 (23.4%; nine participants in up-titration scheme 2 and 10 in up-titration scheme 4) had ARIA-E MRI findings and 15 (18.5%; eight participants in up-titration scheme 2, one in up-titration scheme 3, and six in up-titration scheme 4) participants had ARIA-H MRI findings.

The median time to first ARIA-E MRI finding was 19.4 weeks, and all the first ARIA-E MRI findings occurred in the first 52 weeks of the OLE (Table 5). Most of the ARIA-E MRI findings in the OLE (81%) had resolved by 24 weeks, and 45% resolved within 12 weeks. While a comparison cannot be made between carriers and noncarriers due to the study design (e.g., different titration regimen), there was no significant difference between heterozygotes and homozygotes on time to first ARIA-E event or to median resolution time.

**Table 5:** Time to first ARIA-E event and time to ARIA-E resolution by *APOE* *ɛ*4 carrier status

	*APOE* *ɛ*4 carrier status				
	1*ɛ*4 (*n* = 100)	2*ɛ*4 (*n* = 38)	Carriers (*n* = 138)	Noncarriers (*n* = 81)	Total (*N* = 219)
**Time to first ARIA-E event**
*n*	28	23	51	19	70
Mean, weeks (SD)	25.12 (11.08)	20.22 (10.15)	22.91 (10.85)	21.68 (11.02)	22.58 (10.83)
Median, weeks	24.29	15.29	21.14	18.43	19.43
Q1–Q3, weeks	14.79–33.93	13.29–26.00	13.71–32.14	14.29–25.71	14.00–26.14
Min–Max, weeks	10.0–45.3	5.1–48.3	5.1–48.3	5.1–46.3	5.1–48.3
**Time to ARIA-E resolution**
Duration of all resolved ARIA-E events
*n*	25	22	47	15	62
Mean, weeks (SD)	19.26 (13.91)	16.95 (11.24)	18.18 (12.65)	19.92 (19.27)	18.60 (14.37)
Median, weeks	16.29	14.93	15.43	13.14	15.36
Q1–Q3, weeks	8.71–22.57	8.29–22.71	8.29–22.71	8.00–20.71	8.14–22.57
Min-Max, weeks	3.3–61.4	3.3–46.7	3.3–61.4	4.9–74.4	3.3–74.4
Duration of all unresolved ARIA-E events
*n*	4	2	6	4	10
Mean, weeks (SD)	51.07 (53.54)	30.43 (8.08)	44.19 (42.97)	25.46 (26.82)	36.70 (36.87)
Median, weeks	34.93	30.43	33.64	17.86	29.93
Q1–Q3, weeks	18.50–83.64	24.71–36.14	24.71–38.71	5.50–45.43	7.00–38.71
Min–Max, weeks	5.9–128.6	24.7–36.1	5.9–128.6	4.0–62.1	4.0–128.6

*APOE* *ɛ*4, apolipoprotein E *ɛ*4 allele; ARIA-E, amyloid-related imaging abnormalities-edema; Q1, first quartile 1; Q3, third quartile; SD, standard deviation.

### Symptoms that were temporally associated with ARIA-E MRI findings

Two approaches to identify symptoms associated with ARIA-E in the OLE were used. The first was a protocol-defined approach, whereby participants were contacted by the investigator within 1 week prior to each MRI, to determine, by nondirective, unbiased questioning, if they experienced any CNS AEs. Such AEs were to be specifically designated in the electronic case report form. Those CNS AEs that were recorded accordingly and were associated with a prospective ARIA-E MRI finding were used for this analysis. Based on this approach, three participants reported three AEs (dizziness, somnolence, and headache) that were considered symptoms associated with an ARIA-E (Table 6). All reported AEs were nonserious; mild or moderate in intensity; and were assessed by the investigator to be related to the study treatment. None of these events led to permanent discontinuation of study treatment.

**Table 6:** AEs reported as symptoms of ARIA-E per study protocol approach (safety-evaluable population with one post-baseline MRI in OLE)

	Total (*N* = 291)
Total number of participants with at least one AE reported as symptoms of ARIA-E (eCRF), *n* (%)	3 (1.4)
Total number of AEs reported as symptoms of ARIA (eCRF), *n*	3
Dizziness, *n* (%)	1 (0.5)
Headache, *n* (%)	1 (0.5)
Somnolence, *n* (%)	1 (0.5)

AE, adverse event; ARIA, amyloid-related imaging abnormalities; ARIA-E, amyloid-related imaging abnormalities-edema; eCRF, electronic case report form; MRI, magnetic resonance imaging; OLE, open-label extension.

The second approach was a *post hoc* analysis. Please refer to the Supplementary Material for further details (Supplementary Table 5).

### Injection site reactions

Of the 225 participants in the OLE, injection site reactions were reported in 92 (40.9%) participants, which included 73 (32.4%) participants with injection site erythema, 22 (9.8%) participants with injection site pruritus, 17 (7.6%) participants with injection site swelling, and 12 (5.3%) participants with injection site induration. In total, 772 events of injection site reaction were reported (Table 7). All events of injection site reaction were nonserious and mild in intensity, except for one case, which was of severe intensity. All events except three resolved, and only 8.6% of participants (eight out of the 92) who experienced an injection site reaction received treatment. No participants withdrew from study treatment due to an injection site reaction.

**Table 7:** Signs and symptoms associated with injection site reactions in the OLE by previous randomization (safety-evaluable population)

	Participants previously on placebo (*n* = 117)	Participants previously on gantenerumab (*n* = 108)	Total (*N* = 225)
Total number of participants with at least one sign or symptom associated with administration-related reactions, *n* (%)	48 (41.0)	44 (40.7)	92 (40.9)
Overall total number of events reported as a sign or symptom associated with administration-related reactions, *n*	377	395	772
General disorders and administration site conditions
Injection site erythema, *n* (%)	41 (35.0)	32 (29.6)	73 (32.4)
Injection site pruritus, *n* (%)	10 (8.5)	12 (11.1)	22 (9.8)
Injection site swelling, *n* (%)	11 (9.4)	6 (5.6)	17 (7.6)
Injection site induration, *n* (%)	9 (7.7)	3 (2.8)	12 (5.3)
Injection site rash, *n* (%)	3 (2.6)	5 (4.6)	8 (3.6)
Injection site pain, *n* (%)	4 (3.4)	3 (2.8)	7 (3.1)
Injection site bruising, *n* (%)	1 (0.9)	1 (0.9)	2 (0.9)
Injection site dermatitis, *n* (%)	1 (0.9)	1 (0.9)	2 (0.9)
Injection site discoloration, *n* (%)	1 (0.9)	1 (0.9)	2 (0.9)
Injection site hematoma, *n* (%)	1 (0.9)	1 (0.9)	2 (0.9)
Injection site inflammation, *n* (%)	2 (1.7)	0	2 (0.9)
Injection site edema, *n* (%)	1 (0.9)	1 (0.9)	2 (0.9)
Injection site urticaria, *n* (%)	1 (0.9)	1 (0.9)	1 (0.4)
Injection site warmth, *n* (%)	2 (1.7)	0	1 (0.4)
Infusion site inflammation, *n* (%)	1 (0.9)	0	1 (0.4)
Infusion site pain, *n* (%)	0	1 (0.9)	1 (0.4)
Injection site hemorrhage, *n* (%)	1 (0.9)	0	1 (0.4)
Injection site irritation, *n* (%)	1 (0.9)	0	1 (0.4)
Injection site macule, *n* (%)	0	1 (0.9)	1 (0.4)
Injection site reaction, *n* (%)	0	1 (0.9)	1 (0.4)
Skin and subcutaneous tissue disorders
Pruritus, *n* (%)	1 (0.9)	0	1 (0.4)
Rash erythematous, *n* (%)	1 (0.9)	0	1 (0.4)

OLE, open-label extension.

### Assessment of changes in amyloid load over time

Amyloid PET burden decreased substantially for all participants with available PET data in the OLE. The group mean Centiloid value after 104 weeks of treatment in the OLE was below the positivity threshold of 24 Centiloids, which is considered to be the threshold for differentiating no to sparse plaques from moderate to frequent plaques in histopathology-verified PET studies.^28^ Data from the amyloid PET substudy for change in Aβ plaque burden from OLE baseline to Week 52, Week 104, and Week 156 assessed using florbetapir ^18^F PET have been previously published.^17,29^

### Efficacy outcomes

Cognition and function as assessed by ADAS-Cog13, Alzheimer’s Disease Cooperative Study-Activities of Daily Living (ADCS-ADL) total score, Clinical Dementia Rating – Sum of Boxes (CDR-SB), and Mini-Mental State Examination (MMSE) in the double-blind part of the study (Supplementary Table 6), and by CDR-SB, Clinical Dementia Rating – Global Score (CDR-GS), MMSE, ADAS-Cog13, and ADCS-ADL in the OLE (Supplementary Table 7, Supplementary Table 8) are reported in the Supplementary Material.

### PK outcomes

Summary statistics of gantenerumab plasma concentrations are reported in Supplementary Table 9.

## DISCUSSION

The purpose of the double-blind, placebo-controlled MR study was to establish efficacy and safety of gantenerumab in participants with mild AD. However, following the preplanned futility analysis of the SR study,^6^ recruitment to the MR study was halted while dosing continued, and enrolled participants were offered the opportunity to join an OLE study. Formal analyses of the primary and secondary endpoints for the double-blind study were not performed, and, due to the conversion to an OLE study, these objectives became exploratory. Overall, gantenerumab at low doses of 105 mg or 225 mg had an acceptable safety and tolerability profile.

The MR study was converted into an OLE to investigate the long-term safety and tolerability of a higher dose of 1,200 mg SC Q4W gantenerumab achieved with the implementation of four up-titration regimens. Overall, gantenerumab doses of up to 1,200 mg SC Q4W had an acceptable safety and tolerability profile in qualifying participants from the double-blind part of MR. Amyloid burden decreased substantially over time, demonstrating a strong pharmacodynamic effect of gantenerumab 1,200 mg SC Q4W. ARIA-E incidence in each of the up-titration regimens was acceptable and lower than expected without up-titration.^21^ There was a relationship between *APOE* *ɛ*4 carrier status and ARIA incidence, with *ɛ*4 homozygotes exhibiting the highest incidence of between 55% and 66% depending on the titration schedule. Most participants did not experience ARIA-E during the study period. When ARIA-E findings did occur, these were mostly asymptomatic. If symptoms were associated with ARIA-E findings, they were mostly nonserious, mild, and were clinically manageable by protocol-defined MRI monitoring and dose intervention. Injection site reactions were nonserious and generally mild in intensity, except for one that was of severe intensity. These findings support and provide evidence of a favorable safety profile of gantenerumab at doses of 1,200 mg administered SC Q4W. To place the ARIA observations in context, ARIA rates in the MR study were comparable with other anti-Aβ monoclonal antibodies at that time; for example, the ENGAGE/EMERGE trials of aducanumab reported an overall ARIA-E incidence of 35% (43% carriers, 20% noncarriers), and the GRADUATE I and GRADUATE II trials of gantenerumab reported an overall ARIA-E incidence of 25% (31% carriers, 13% noncarriers), compared with 32% (37% carriers, 24% noncarriers) in MR.^12,30^ The slower titration used in the GRADUATE studies resulted in the reduction in the overall incidence of ARIA-E in carriers in *APOE* *ɛ*4 homozygotes.^31^

The results of the MR OLE study should be interpreted in the context of the following limitations. First, there was no placebo arm in the MR OLE; thus, no conclusions regarding the clinical efficacy of gantenerumab can be drawn. However, an exploratory analysis comparing the MR OLE (and SR OLE) data with an Alzheimer’s Disease Neuroimaging Initiative external control showed a slowing of cognitive decline compared with these external controls, as seen on CDR-SB and other measures, following continued gantenerumab treatment over 3 years.^32^ Second, the titration regimens were confounded by baseline risk factors and speed of up-titration, due to which comparisons between the different titration regimens cannot be made.

In summary, data from the MR OLE study provide evidence of a favorable benefit-risk profile of SC gantenerumab at this higher (1,200 mg SC Q4W) target dose. With this overall profile, participants who completed the OLE of MR (along with participants who completed the OLE of SR,^23^ [Boada M et al. unpublished data]) were given the option to continue open-label use of gantenerumab in the Open RoAD study (NCT04339413), an open-label, multicenter, rollover study to evaluate the safety and tolerability of long-term administration of gantenerumab.^33^ Following the negative preplanned analysis of pivotal Phase III studies GRADUATE I and GRADUATE II (see below), Open RoAD was terminated by the sponsor, as the benefit-risk assessment in participants with AD was no longer considered favorable.

MR OLE data were used to inform the design of the pivotal Phase III GRADUATE I and GRADUATE II studies, which included a single 9-month titration regimen that aimed to maximize exposure to SC gantenerumab for all participants, regardless of *APOE* *ɛ*4 status.^31^ The outcomes from the GRADUATE studies indicated a lower overall effect on pharmacodynamic measures, with notably lower amyloid plaque removal than observed in the SR and MR OLE studies,^28,29^ and statistically significant clinical efficacy was not demonstrated.^24^ The reasons for these outcomes are currently beingexplored.

## AUTHOR CONTRIBUTIONS

Anuja Neve (Data curation; Formal analysis; Supervision; Writing – original draft; Writing – review & editing; Approval of final version of manuscript for submission); Bibha Das (Data curation; Formal analysis; Writing – review & editing; Approval of final version of manuscript for submission); Jakub Wojtowicz (Data curation; Formal analysis; Writing – review & editing; Approval of final version of manuscript for submission); Zhiyue Huang (Data curation; Formal analysis; Writing – review & editing; Approval of final version of manuscript for submission); Szofia Bullain (Formal analysis; Writing – review & editing; Approval of final version of manuscript for submission); Michelle Watkin (Formal analysis; Writing – review & editing; Approval of final version of manuscript for submission); Dominik Lott (Formal analysis; Writing – review & editing; Approval of final version of manuscript for submission); Tobias Bittner (Formal analysis; Writing – review & editing; Approval of final version of manuscript for submission); Paul Delmar (Formal analysis; Writing – review & editing; Approval of final version of manuscript for submission); Gregory Klein (Formal analysis; Writing – review & editing; Approval of final version of manuscript for submission); Carsten Hofmann (Conceptualization; Formal analysis; Methodology; Writing – review & editing; Approval of final version of manuscript for submission); Geoffrey A. Kerchner (Formal analysis; Writing – review & editing; Approval of final version of manuscript for submission); Janice Smith (Formal analysis; Writing – review & editing; Approval of final version of manuscript for submission); Monika Baudler (Formal analysis; Writing – review & editing; Approval of final version of manuscript for submission); Paulo Fontoura (Conceptualization; Formal analysis; Supervision; Writing – review & editing; Approval of final version of manuscript for submission); Rachelle S. Doody (Formal analysis; Writing – review & editing; Approval of final version of manuscript for submission).

## Supplementary Material

Supplementary Material

## Data Availability

For eligible studies, qualified researchers may request access to individual participant-level clinical data through a data request platform. At the time of writing, this request platform is Vivli (https://vivli.org/ourmember/roche/). For up-to-date details on Roche’s Global Policy on the Sharing of Clinical Information and how to request access to related clinical study documents, see here: https://go.roche.com/data_sharing. Anonymized records for individual participants across more than one data source external to Roche cannot, and should not, be linked, due to a potential increase in risk of patient re-identification.
