# Supplementary material for: Long-Term Safety of Gantenerumab in Participants with Alzheimer’s Disease: A Phase III, Double-Blind, and Open-Label Extension Study (Marguerite RoAD)
Source: J Alzheimers Dis. 2024 Aug 27;101(1):353–67. doi: 10.3233/JAD-240221 (PMC11380290; doi:10.3233/JAD-240221)
Supplement: Supplementary Material [file jad-101-jad240221-s001.pdf]

Supplementary Material

Long-Term Safety of Gantenerumab in Participants with Alzheimer's Disease: A Phase III, Double-Blind, and Open-Label Extension Study (Marguerite RoAD)

Contents

Supplementary methods 2
Outcome measures in the double-blind part 2
Management of MRI findings in the double-blind part 2
Statistical analyses in the double-blind part 2
Supplementary results 3
Study population in the double-blind part 3
Baseline characteristics in the double-blind part 3
Concomitant medications in the double-blind part and OLE 3
Summary of AEs in the double-blind part 4
Incidence of ARIA MRI findings in the double-blind part 4
Symptoms that were associated with ARIA-E MRI findings in the double-blind part and OLE 4
Cognition and function 5
Assessment of changes in amyloid load over time in the double-blind part 6
Pharmacokinetics (PK) 6
Exploratory biomarkers in the double-blind part 6
Supplementary tables ..... **Error! Bookmark not defined.**
Supplementary Table 1 Complete inclusion and exclusion criteria for the double-blind part and OLE 7
Supplementary Table 2 Schedule of assessments in (a, b) the MR double-blind part and (c–g) the OLE 10
Supplementary Table 3 AEs occurring in ≥5% of participants in the OLE, by class and previous randomization 31
Supplementary Table 4 ARIA-E events in the OLE by dosing step, titration regimen, and *APOE* ε4 status (safety-evaluable population with post-baseline MRI in the OLE) 32
Supplementary Table 5 AEs associated with ARIA-E MRI findings and retrieved using a post hoc programmatic approach (safety-evaluable participants with post-baseline MRI in OLE) 34
Supplementary Table 6 Summary of efficacy results and change from baseline values to Week 104 using MMRM analysis in double-blind part 35
Supplementary Table 7 Summary of efficacy outcomes and change from OLE baseline at Week 104 by previous randomization (treated OLE participants) 36
Supplementary Table 8 MMSE total scores and change from OLE baseline by visit past Week 104 by previous randomization (safety-evaluable population) 37
Supplementary Table 9 Summary statistics of gantenerumab plasma concentrations per scheduled visit in the OLE 38

SUPPLEMENTARY METHODS

Outcome measures in the double-blind part

The primary objective in the double-blind part was to evaluate the efficacy of gantenerumab given at doses of 105 or 225 mg subcutaneously (SC) every 4 weeks (Q4W) from baseline to Week 104, as measured by two co-primary endpoints: cognition (Alzheimer's Disease Activity Scale-Cognitive subscale 13 [ADAS-Cog 13]) and function (Alzheimer's Disease Cooperative Study-Activities of Daily Living [ADCS-ADL]).

The key secondary efficacy endpoints in the double-blind part included clinical decline and disease progression by assessment of Mini-Mental State Exam (MMSE), ADCS-ADL instrumental activities of daily living (IADL), Clinical Dementia Rating scale (CDR)-Sum of Boxes (CDR-SB) and ADAS-Cog 13 responders at Week 104.

As the recruitment was halted and the study was converted to an open-label extension (OLE), all efficacy objectives, including the co-primary and secondary endpoints, became exploratory, given the reduced number of participants in the study and the fact that there were few participants who had completed the double-blind treatment period.

Additional secondary outcomes included the change from baseline to Week 104 in disease pathology biomarkers, assessed using cerebrospinal fluid (CSF) amyloid-β $(A\beta)_{42}$, total tau (t-tau) and phosphorylated tau (p-tau). Behavior was assessed using the Neuropsychiatric Inventory Questionnaire (NPI-Q).

Safety and tolerability were assessed by magnetic resonance imaging (MRI), physical and neurologic examinations, vital signs, blood safety tests, electrocardiograms, Columbia Suicide Severity Rating Scale and adverse event (AE) monitoring.

Management of MRI findings in the double-blind part

In the case of a single amyloid-related imaging abnormalities (ARIA)-edema (ARIA-E) >2 cm or multiple ARIA-E, dosing was stopped until the ARIA-E had resolved or significantly decreased or stabilized. For single cases of ARIA-E ≤2 cm, no change of dosing was required. For participants on 225 mg gantenerumab who developed two new microhemorrhage on a single scan or 3–4 new microbleeds cumulatively, the dose was reduced to 105 mg. Participants who developed five or more new microbleeds cumulatively were discontinued from the study treatment.

Statistical analyses in the double-blind part

As a result of the recruitment being halted and the study being converted to an OLE, exploratory and descriptive statistics were applied to all endpoints in the double-blind part of the study. For participants who did not transition into the OLE, this included all data from all visits up to and including the final follow-up visit (Week 152). For participants who

transitioned into the OLE, data from all visits up to and including the Week 104/early termination visit (Follow-up 1), or the last visit prior to first OLE dose, were included. No data from the OLE were used. Time windowing was used to ensure that data from all Week 104/ early termination visits were summarized at the correct timepoint.

SUPPLEMENTARY RESULTS

Study population in the double-blind part

By November 2015, when recruitment for Marguerite RoAD (MR) had been suspended, a total of 389 participants had enrolled in the double-blind part of the study, of whom, 387 were treated. The disposition of participants in the double-blind and OLE parts is available in Fig. 3. Overall, 195 participants received placebo and 192 received gantenerumab. Sixty-one participants (31.3%) receiving placebo and 56 participants (29.2%) receiving gantenerumab withdrew from the study before the OLE. The most common reason for discontinuation was participant withdrawal (placebo: 21.5%; gantenerumab: 15.1%). Study withdrawals due to AEs occurred in 1.0% of those on placebo and 4.2% of those on gantenerumab.

Baseline characteristics in the double-blind part

Participants had a mean (standard deviation [SD]) age at baseline of 70.1 (8.6) years among those treated with placebo and 69.7 (8.9) among those treated with gantenerumab; most were female (placebo: 57.9%; gantenerumab: 51.0%) and White (placebo: 84.1%; gantenerumab: 86.5%). Among those who received placebo and gantenerumab, the apolipoprotein E (*APOE*) 1ε4 genotype was the most common (48.7% and 44.8%, respectively), followed by 0ε4 (32.8% and 34.4%, respectively) and 2ε4 (18.5% and 20.8%, respectively). Mean (SD) duration of Alzheimer's disease (AD) was comparable between those treated with placebo (23.4 [22.2] months) and gantenerumab (23.9 [23.0] months). Twenty-two participants (13 participants on placebo and nine participants on gantenerumab) completed double-blind treatment in the study.

Concomitant medications in the double-blind part and OLE

In the double-blind part of the study, 15 (2.1%) participants reported use of memantine; 40 (10.3%) participants reported the use of cholinesterase inhibitors (rivastigmine, donepezil, galantine). After OLE baseline, seven (3.1%) participants reported use of memantine, five (2.2%) participants reported use of acetylcholinesterase inhibitors (donepezil or galantamine) and one (0.4%) reported the use of combination (memantine and donepezil).

Summary of AEs in the double-blind part

In the double-blind part, the mean (SD) treatment duration for participants who received gantenerumab was 65.44 (25.60) weeks. Among participants who received placebo, the mean treatment duration was 66.72 (26.58) weeks. Most participants in each treatment group experienced at least one AE (80.5% of participants on placebo and 82.8% on gantenerumab). The most common (>7%) AEs were fall (7.7% on placebo, 9.4% on gantenerumab), nasopharyngitis (7.7% on placebo, 7.3% on gantenerumab) and headache (7.7% on placebo, 6.3% on gantenerumab). Thirteen participants (6.8%) who received gantenerumab and five participants (2.6%) who received placebo had AEs that led to withdrawal from the study treatment. Nineteen participants (9.9%) on gantenerumab and 11 participants (5.6%) on placebo had AEs leading to dose modification of study treatment. Twenty-three participants (12.0%) on gantenerumab and 24 participants (12.3%) on placebo reported serious AEs. The most frequent (>0.5%) serious AEs were fall (1.3%, five participants [1.5% on placebo and 1.0% on gantenerumab]) and atrial fibrillation (0.5%, two participants [0.5% on placebo and 0.5% on gantenerumab]). There were three deaths in the gantenerumab group (cardiac arrhythmia, cardiac arrest and fall) and three deaths in the placebo group (cardiac failure, ovarian epithelial cancer and cerebrovascular accident); all were considered unrelated to study treatment by the study investigator. There were no AEs of special interest reported (drug-induced liver injury, suspected transmission of infectious agent) and no clinically significant safety findings related to physical exams, vital signs, laboratory test results or electrocardiogram data.

Incidence of ARIA MRI findings in the double-blind part

In the double-blind part, the incidence of ARIA-E AEs was higher in those who received gantenerumab (9.4% [18 participants]) than in those who received placebo (1.5% [three participants]). The incidence of ARIA-hemorrhage (ARIA-H) AEs was 6.3% (12 participants) in those who received gantenerumab and 4.1% (eight participants) in those who received placebo.

Symptoms that were associated with ARIA-E MRI findings in the double-blind part and OLE

In the double-blind part, participants were asked up to 1 week before each MRI was performed if they had experienced central nervous system (CNS) AEs. This approach identified two AEs (irritability and headache) that were considered symptoms of ARIA-E and were temporally associated with an ARIA-E. Irritability experienced in one participant was assessed by investigator to be not related to the study treatment (placebo), whereas the headache was assessed as related to the study treatment (gantenerumab).

In the OLE, two approaches to identifying symptoms associated with ARIA-E were used. The first was a protocol-defined approach, which is described in the main manuscript. The second approach was a post hoc analysis, whereby AEs associated with ARIA-E MRI findings were retrieved programmatically. These were defined as AEs of nervous system disorders or psychiatric disorders that had an onset within 4 weeks prior to ARIA-E onset, through to the ARIA-E resolution date. AEs of ARIA MRI findings were excluded from this analysis. Based on this approach, of the 219 participants who received gantenerumab and had a post-baseline MRI, 22 (10.0%) participants reported at least one CNS AE that was temporally associated with an ARIA-E MRI finding; a total of 33 CNS AEs were reported, the most common of which were headache (1.8%), dizziness (1.4%) and syncope (1.4%) (Supplementary Table 5). CNS symptoms associated with ARIA-E MRI findings were mostly mild to moderate in intensity; five required permanent discontinuation of study treatment (cognitive disorder, confused state, generalised tonic-clonic seizure, white matter lesion and hemiplegia). Three CNS AEs were reported as serious (two seizures and one hemiplegia); two of these three serious AEs were considered related to study treatment.

Cognition and function

In the double-blind part, treatment with gantenerumab did not lead to a statistically significant difference compared with placebo in change from baseline to Week 104 in ADAS-Cog 13 scores (mean difference: −3.71 [95% confidence interval [CI]: −7.54, 0.12]; p=0.058) or ADCS-ADL (mean difference: 3.84 [95% CI: −1.29, 8.97]; p=0.140); however, as the recruitment was halted and the study was converted to OLE, all efficacy objectives became exploratory. Additional efficacy data for both the double-blind and OLE stages can be found in Supplementary Table 6, Supplementary Table 7, and Supplementary Table 8.

In the OLE, for the 117 participants previously on placebo, the mean (SD) change from OLE baseline at Week 104 for CDR-SB was 8.33 (3.67), for CDR – Global Score was 1.39 (0.60), for MMSE was 16.65 (6.59), for ADAS-Cog 13 was 43.80 (15.76), and for ADCS-ADL was 49.76 (17.04).

The mean change (SD) from OLE baseline for MMSE total score at Week 180 was –7.55 (5.43), at Week 208 was –9.48 (3.96), and at Week 232 was –9.43 (5.24). For the 108 participants previously on gantenerumab, the mean (SD) change from OLE baseline at Week 104 for CDR-SB was 4.19 (3.71), for CDR – Global Score was 0.69 (0.78), for MMSE was –5.96 (5.30), for ADAS-Cog 13 was 13.38 (15.41), and for ADCS-ADL was –14.78 (16.70). The mean change (SD) from OLE baseline for MMSE total score at Week 180 was –8.07 (5.64), at Week 208 was –7.59 (5.81), and at Week 232 was –10.43 (6.72).

Assessment of changes in amyloid load over time in the double-blind part

A total of 112 participants (61 on placebo and 51 on gantenerumab) had a baseline PET scan in the double-blind phase of the study. No significant change in amyloid load was observed with treatment with gantenerumab compared with placebo; however, numeric decreases in cortical composite standardized uptake value ratios (SUVRs) (reference region: cerebellum gray) were observed in those treated with gantenerumab compared with placebo at Weeks 48 and 104.

Pharmacokinetics (PK)

In the OLE, a total of 1,453 (five prior to first OLE dosing, 1,448 following first dosing) gantenerumab plasma concentration samples from 223 participants were collected over a treatment period of 208 weeks. Different dose escalation schedules based on participants' *APOE* ε4 carrier status (carrier vs noncarrier) and the treatment received during the double-blind part of the study (105 mg gantenerumab, 225 mg gantenerumab, or placebo) were used to reach the target dose of 1,200 mg gantenerumab Q4W. For this reason, gantenerumab doses and PK sampling schedules were different during the first 6 months of the OLE. Following Week 28, all participants were to receive doses of 1,200 mg gantenerumab Q4W, and PK and plasma concentrations were measured at Weeks 53, 101, 104, 116, 156, and 208. Overall, the observed PK profile was in agreement with the previously observed gantenerumab PK profile at doses of 105 and 225 mg administered in the double-blind part of the study (Supplementary Table 9).

Exploratory biomarkers in the double-blind part

In the double-blind part, treatment with gantenerumab led to a significant decrease from baseline to Week 104 in p-tau levels compared with placebo. For p-tau, the median change from baseline to Week 104 was –16.3% (n = 12) for gantenerumab and 1.41% (n = 20) for placebo. A numerical, but nonsignificant, decrease in t-tau levels was observed in participants treated with gantenerumab when compared with placebo. For t-tau, the median change from baseline to Week 104 was –6.17% (n = 12) for gantenerumab and 3.87% (n = 20) for placebo. No significant change was seen for $A\beta_{42}$ and $A\beta_{40}$ in either treatment group.

Supplementary Table 1

Complete inclusion and exclusion criteria for the double-blind part and OLE

Inclusion criteria	Exclusion criteria
Double-blind part	
• Ability to provide written consent signed by the participant (co-signed by the participant's legally authorized representative, if required by the local regulations, guidelines, and IEC or IRB)<br>• Aged 50–90 years<br>• Clinical diagnosis of probable mild AD based on NINCDS/ADRDA criteria or major neurocognitive disorder due to AD of mild severity based on the DSM-5 criteria whether or not receiving AD approved medication<br>• If the participant was receiving AD medications, the dosing regimen must have been stable for 3 months prior to screening<br>• For people of non-childbearing potential (more than 2 years after the cessation of menses or surgically sterile by means of hysterectomy, bilateral oophorectomy, or tubal ligation), additional blood or urine tests were performed for further confirmation of non-childbearing potential, if required by local regulations, guidelines and IRB/IEC<br>• For people of childbearing potential, a negative urine ß-hCG was required at screening and baseline, and agreement to use two acceptable forms of effective contraception from the screening visit until 16 weeks after study drug discontinuation<br>• Availability of a caregiver who has frequent and sufficient contact with the participant; was able to provide accurate information regarding the participant's cognitive and functional abilities; agreed to provide information at clinic visits, which required partner input for scale completion; and signed the necessary consent form<br>• Fluency in the language of the tests used at the study site	• Neurological disease other than AD, a major psychiatric disorder, a history of stroke or any clinically unstable medical illness<br>• MRI evidence of:<br>a) >2 lacunar infarcts;<br>b) any territorial infarct >1 cm^3;<br>c) any white matter lesion corresponding to an overall Fazekas score of 3 that requires at least one confluent hyperintense lesion on the FLAIR sequence, which was ≥20 mm in any dimension;<br>d) combined number of microbleeds and areas of leptomeningeal hemosiderosis >4 on a 1.5-T machine or >5 on a 3-T machine, or;<br>e) presence of any other significant cerebral abnormalities, including ARIA-E<br>• Any unstable medical condition or history of clinically significant cardiovascular disease, hepatic renal disorders, infections, immune disorders, metabolic and endocrine disorders, and cancer<br>• Intellectual disability<br>• Contraindications of a lumbar puncture<br>• Clinically significant abnormal screening blood, urine, or CSF that remained abnormal on retest<br>• Screening prothrombin time > 1.2 x the upper limit of normal<br>• Hypersensitivity to any of gantenerumab's excipients<br>• Participants who required residence in a skilled nursing facility<br>• Previous administration of immunotherapy to prevent or delay cognitive decline, investigational treatment, medications to treat other neurodegenerative disorders

• Willingness and ability to complete all aspects of the study (including MRI, lumbar puncture, clinical genotyping, and PET imaging, if applicable); the participant should be capable of completing assessments either alone or with the help of the caregiver<br>• Adequate visual and auditory acuity, in the investigator's judgment, sufficient to perform the neuropsychological testing (eye glasses and hearing aids are permitted)<br>• Agreement not to donate blood or blood products for transfusion for the duration of the study and for 1 year after final dose of study drug<br>• Agreement not to participate in other research studies for the duration of this trial and its associated substudies<br>• Screening MMSE score of 20–26 points, inclusive, at screening<br>• Screening CDR-GS of 0.5–1.0<br>• Screening Geriatric Deterioration Scale-15 score <6<br>• CSF Aß$_{42}$ levels <700 pg/mL, as measured on the Elecsys® assay	(>1 year of screening), and other concomitant medications
OLE	
• Participants with mild AD who were enrolled in the double-blind part of MR<br>• All participants who had been randomized and actively participated in the study at the time of the protocol amendment (Version 4) approval in their respective country were eligible to participate in the OLE<br>• Age ≥50 to ≤90 years, with a clinical diagnosis of probable mild AD based on NINCDS/ADRDA criteria or probable neurocognitive disorder due to AD, mild severity based on the DSM-5 criteria<br>• Participants were eligible regardless of whether they were receiving approved AD medications. If the participant was receiving AD medications, the dosing regimen must have been stable for 3 months prior to screening	• Neurological disease other than AD, a major psychiatric disorder, a history of stroke or any clinically unstable medical illness<br>• MRI evidence of:<br>a) >2 lacunar infarcts;<br>b) any territorial infarct >1 cm^3;<br>c) any white matter lesion corresponding to an overall Fazekas score of 3 that requires at least one confluent hyperintense lesion on the FLAIR sequence, which was ≥20 mm in any dimension;<br>d) combined number of microbleeds and areas of leptomeningeal hemosiderosis >4 on a 1.5-T machine or >5 on a 3-T machine, or;<br>e) presence of any other significant cerebral abnormalities, including ARIA-E

• Having the following cognition-related and test-based criteria for mild AD:<br>a) Screening MMSE score of 20−26 points, inclusive<br>b) Screening CDR-GS of 0.5−1.0<br>c) Screening Geriatric Depression Scale-15 score <6<br>d) CSF $A\beta_{42}$ levels ≤700 pg/mL on the Elecsys® assay	

Aβ, amyloid-β; AD, Alzheimer's disease; ADRDA, Alzheimer's Disease and Related Disorders Association; ARIA-E, amyloid-related imaging abnormalities – edema; ß-hCG, beta-human chorionic gonadotropin; CDR-GS, Clinical Dementia Rating Scale – Global Score; CSF, cerebrospinal fluid; DSM-5, Diagnostic and Statistical Manual of Mental Disorders, Version 5; FLAIR, fluid-attenuated inversion recovery; IEC, independent ethics committee; IRB, institutional review board; MMSE, Mini-Mental State Exam; MRI, magnetic resonance imaging; MR, Marguerite RoAD; NINCDS, National Institute of Neurological and Communicative Disorders and Stroke; OLE, open-label extension; PET, positron emission tomography.

Supplementary Table 2

Schedule of assessments in (a, b) the MR double-blind part and (c–g) the OLE

a) Double-blind Year 1

Assessment/ procurement	Screening	BL	Treatment period														Unsch[a]
	Wk 1 to Wk 18	Day 1	Day 4 (± 2 days)	Wk 4	Wk 8	Wk 12	Wk 16	Wk 20	Wk 24	Wk 28	Wk 32	Wk 36	Wk 40	Wk 44	Wk 48	Wk 52	
Dose number		1		2	3	4	5	6	7	8	9	10	11	12	13	14	
Informed consent(s)[b]	x																
Review of inclusion and exclusion criteria	x																
Medical history, personal status, and demographics[c]	x																
Physical exam	x														x		x
Neurologic exam	x														x		x
Clinical genotyping sample	x[d]																
RCR DNA sample[e]		x															
RCR RNA sample[e]		x															
RCR plasma sample [e]		x															
Vital signs[f]	x[f]	x[f]		x	x	x	x	x	x	x	x	x	x	x	x	x	x[f]
Twelve-lead ECG[g]	x	x													x		x
Serum chemistry[h] and hematology[i]	x[h]	x							x						x[h]		x
PK plasma sample[j]		x	x	x	x	x			x						x		x
Anti-drug antibody sample		x[j]		x	x	x			x						x		x
Coagulation (prothrombin time)	x																x

Urine sample for drugs of abuse	x																x
Urinalysis[k]	x																x
Urine pregnancy test[l]	x	x[l]		x[l]	x[l]	x[l]	x[l]	x[l]	x[l]	x[l]	x[l]	x[l]	x[l]	x[l]	x[l]	x[l]	x
Lumbar puncture[m,n] and CSF sampling[n,o]	x[n,o]															x[o]	x
MRI scan[p]	x[n,q,r]								x[q,r]			x			x[q,r]		x
Adverse event	x	x	x	x	x	x	x	x	x	x	x	x	x	x	x	x	x
Concomitant meds	x	x	x	x	x	x	x	x	x	x	x	x	x	x	x	x	x
ADAS-Cog 13	x	x							x						x		x
CDR[s]	x	x							x						x		x
ADCS-ADL[s]	x	x							x			x			x		x
MMSE	x	x							x			x			x		x
NPI[s]		x							x						x		x
GDS	x																x
QoL-AD		x							x						x		x
ZCI-AD[s]		x													x		x
Dependence scale[s]		x													x		x
SymptomGuide™ Facilitated GAS (if applicable)[s,t]		x			x				x						x		x
C-SSRS		x		x	x	x	x	x	x	x	x	x	x	x	x	x	x
RUD-Lite[s]		x															x
Study drug administration[u]		x		x	x	x	x	x	x	x	x	x	x	x	x	x	

ADAS-Cog 13, Alzheimer's Disease Activity Scale – Cognitive Subscale 13; ADCS-ADL, Alzheimer's Disease Cooperative Study – Activities of Daily Living; ALT, alanine transaminase; *APOE* ε4, apolipoprotein E ε4 allele; AST, aspartate aminotransferase; BL, baseline; BP, blood pressure; BUN, blood urea nitrogen; CDR, Clinical Dementia Rating; CSF, cerebrospinal fluid; C-SSRS, Columbia Suicide Severity Rating Scale; ECG, electrocardiogram; GAS, Goal Attainment Scaling; GDS, Geriatric Deterioration Scale; HR, heart rate; MMSE, Mini-Mental State

Examination; MR, Marguerite RoAD; MRI, magnetic resonance imaging; NPI, Neuropsychiatric Inventory; OLE, open-label extension; PET, positron emission tomography; PK, pharmacokinetic; QoL-AD, quality of life in Alzheimer's disease; RCR, Roche Clinical Repository; RUD-Lite, Resource Utilization Dementia – Lite version; SC, subcutaneous; SGOT, serum glutamic-oxaloacetic transaminase; SGPT, serum glutamate pyruvate transaminase; Unsch, unscheduled; WBC, white blood cell; Wk, Week; ZCI-AD, Zarit Caregiver Interview for Alzheimer's disease.

Note: At the BL visit, all safety assessments must be conducted on the day of dosing and all other assessments may be conducted up to 7 days earlier. On Day 4, the visit window is ± 1 day. The visit window is ± 7 days for all other visits.

[a]For participants who terminate early, the assessments from Wk 104 should be used.
[b]The MR PET substudy and Ccardiac PET substudy are optional, and participants who elect to participate must sign a separate informed consent.
[c]Medical history includes clinically significant diseases, surgeries, cancer history (including prior cancer therapies and procedures), smoking history, use of alcohol and drugs of abuse; and all medications (e.g., prescription drugs, over-the-counter drugs, herbal/homeopathic remedies, nutritional supplements) used by the participant within 6 months prior to the screening visit. Demographics include age, sex, and self-reported race/ethnicity.
[d]At screening, three mandatory 3 mL whole blood samples will be obtained for DNA extraction for analysis of *APOE* ε4 status and Fcγ receptor genotype.
[e]Optional RCR samples for exploratory analysis from consenting participants should be obtained at the same time that the blood samples are obtained.
[f]Vital signs include HR and BP and at screening, Day 1, and unscheduled visits also include body temperature. The same arm should be used for all BP measurements. HR and BP should not be measured unless 15 minutes have passed since the last blood draw. Weight will only be collected at screening and Wk 104 visits.
[g]Perform after the participant has been in a supine position for 5 minutes. ECGs for each participant should be obtained from the same machine, whenever possible, and performed prior to any blood draws, brain MRI scans, and lumbar puncture.
[h]Serum chemistry includes AST/SGOT, ALT/SGPT, alkaline phosphatase, total protein, total bilirubin, serum albumin, creatine phosphokinase, sodium, potassium, calcium, BUN, and serum creatinine (and creatinine clearance calculated by the central laboratory). During the screening period (Wk –1 to Wk –8) and at Wks 48 and 104, hemoglobin A1c, folic acid, vitamin B12, T4, free T4, and thyroid-stimulating hormone levels will also be assessed.
[i]Hematology includes hemoglobin, hematocrit, red blood cell (with morphology), WBCs, platelet, basophil, eosinophil, lymphocyte, monocyte, neutrophil, and WBC–other total counts.
[j]The BL sample will be obtained prior to first dose. All PK samples except the Day 4 sample should be obtained just before administration of study drug (gantenerumab or placebo) or during the specified visits, if possible. Accurate recording of the time of study drug administration and PK sampling is critical.
[k]Urinalysis will be performed at the site by dipstick for blood, protein, glucose, and pH. Microscopic examination performed at the central laboratory if blood and/or protein results are positive or strongly positive.

[l]Females of childbearing potential (including those who have had a tubal ligation) must have a urine pregnancy test performed at the site prior to each dose administration. If a urine pregnancy test is positive, it must be confirmed by a serum pregnancy test at the central laboratory.
[m]Lumbar puncture must be performed in the morning (between 8:00 a.m. and 12:00 p.m.), to minimize potential diurnal variation of CSF parameters. Lumbar puncture should be performed 10–20 days after dosing.
[n]CSF sampling and MRI (and PET scan if the participant is enrolled in any of the PET substudies) at screening should be performed once all other screening results are available and none exclude the participant from the trial. For participants enrolled in any of the PET substudies, PET may be performed after the lumbar puncture and prior to when CSF results are received; there is no requirement for CSF results to be available before the PET. After aliquoting the required samples, any remaining CSF fluid from consenting participants will be kept for future RCR biomarker research.
[o]CSF samples are mandatory at screening; collection at Wk 52 is optional.
[p]MRI should not be performed for 3 days following a lumbar puncture. MRI scans must be performed within a maximum of 20 days after dose administration and results made available and reviewed before the next scheduled dose. The final MRI should be performed approximately 4 weeks before the final follow-up visit, to allow for review before the final visit.
[q]Includes volumetric MRI outcome measures.
[r]Includes functional MRI outcome measures.
[s]Scale requires caregiver input or support.

[t]The SymptomGuide™ Facilitated GAS will be conducted at investigational sites in French- and English-speaking countries.
[u]Study drug administration should be performed only after all assessments/rating scales for the participant are completed (unless indicated otherwise). Study drug will be administered to participants as SC injection to the abdomen. Study personnel administering study drug must not be involved with any efficacy assessments or safety evaluations. Following the first four doses, participants should be observed for a minimum of 2 hours after dosing; for the remaining doses, participants should be observed for a minimum of 1 hour. On days when only safety is being assessed, participants may have the option to have the study drug administered and applicable safety assessments conducted at a prearranged location away from the site by a trained healthcare professional, if consent is obtained.

b) Double-blind Year 2

Assessment/ procurement	Treatment period												Follow-up		Additional follow-up	Unsch[a]
	Wk 56	Wk 60	Wk 64	Wk 68	Wk 72	Wk 80	Wk 84	Wk 84	Wk 88	Wk 92	Wk 96	Wk 100	Wk 104 or early term[a]	Wk 116	Wk 152	
Dose number	15	16	17	18	19	20	21	22	23	24	25	26				
Physical examination													x	x	x	x
Neurologic examination													x	x	x	x
Vital signs[b]	x	x	x	x	x	x	x	x	x	x	x	x	x	x	x	x[b]
12-Lead ECG[c]													x	x	x	x
Serum chemistry[d] and hematology[e,f]					x								x[d]		x	x
PK plasma sample					x								x	x		x
Anti-drug antibody sample					x								x	x		x
Urinalysis[g]																x
Urine pregnancy test[h]	x	x	x	x	x	x	x	x	x	x	x	x				
Lumbar puncture[i,j] and CSF sampling[j,k]													x[k]		x[k]	x
MRI scan[l]													x[m,n]		x[m]	x
Adverse events	x	x	x	x	x	x	x	x	x	x	x	x	x	x	x	x
Concomitant meds	x	x	x	x	x	x	x	x	x	x	x	x	x	x	x	x
CDR[o]					x								x		x	x
ADAS-Cog 13					x								x	x	x	x
ADCS-ADL[o]		x			x			x			x		x	x	x	x
NPI[o]					x								x		x	x

MMSE		x			x			x			x		x	x	x	x
C-SSRS	x	x	x	x	x	x	x	x	x	x	x	x	x	x	x	x
Dependence scale[o]													x		x	x
RUD-Lite[o]													x		x	x
ZCI-AD[o]													x		x	x
QoL-AD					x						x		x		x	x
SymptomGuide™ Facilitated GAS (if applicable)[o,p]					x								x		x	x
Study drug administration[q]	x	x	x	x	x	x	x	x	x	x	x	x				

ADAS-Cog 13, Alzheimer's Disease Activity Scale – Cognitive Subscale 13; ADCS-ADL, Alzheimer's Disease Cooperative Study – Activities of Daily Living; ALT, alanine transaminase; AST, aspartate aminotransferase; BP, blood pressure; BUN, blood urea nitrogen; CDR, Clinical Dementia Rating; CSF, cerebrospinal fluid; C-SSRS, Columbia Suicide Severity Rating Scale; ECG, electrocardiogram; GAS, Goal Attainment Scaling; HR, heart rate; MMSE, Mini-Mental State Examination; MRI, magnetic resonance imaging; NPI, Neuropsychiatric Inventory; PET, positron emission tomography; PK, pharmacokinetic; QoL-AD, quality of life in Alzheimer's disease; RCR, Roche Clinical Repository; RUD-Lite, Resource Utilization Dementia – Lite version; SC, subcutaneous; SGOT, serum glutamic-oxaloacetic transaminase; SGPT, serum glutamate pyruvate transaminase; WBC, white blood cell; Wk, Week; Unsch, unscheduled; ZCI-AD, Zarit Caregiver Interview for Alzheimer's disease.

Note: The visit window is ± 7 days for all visits.

[a]For participants who terminate early, the unscheduled visit assessments should be used.
[b]Vital signs include HR and BP, and at unscheduled visits also include body temperature. The same arm should be used for all BP measurements. HR and BP should not be measured unless 15 minutes have passed since the last blood draw. Weight will only be collected at screening and Wk 104 visits.
[c]Perform after the participant has been in a supine position for 5 minutes. ECGs for each participant should be obtained from the same machine, whenever possible, and performed prior to any blood draws, brain MRI scans, and lumbar puncture.
[d]Serum chemistry includes AST/SGOT, ALT/SGPT, alkaline phosphatase, total protein, total bilirubin, serum albumin, creatine phosphokinase, sodium, potassium, calcium, BUN, and serum creatinine (and creatinine clearance calculated by the central laboratory). At Wk 104, hemoglobin A1c, folic acid, vitamin B12, T4, free T4, and thyroid-stimulating hormone levels will also be assessed.
[e]Hematology includes hemoglobin, hematocrit, red blood cell (with morphology), WBCs, platelet, basophil, eosinophil, lymphocyte, monocyte, neutrophil, and WBC–other total counts.
[f]A PK sample will be obtained at unscheduled visits.

[g]Urinalysis will be performed at the site by dipstick for blood, protein, glucose, and pH. Microscopic examination performed at the central laboratory if blood and/or protein results are positive or strongly positive.
[h]Females of childbearing potential (including those who have had a tubal ligation) must have a urine pregnancy test performed at the site prior to each dose administration. If a urine pregnancy test is positive, it must be confirmed by a serum pregnancy test at the central laboratory.
[i]Lumbar puncture must be performed in the morning (between 8:00 a.m. and noon [12:00 p.m.]), to minimize potential diurnal variation of CSF parameters. Lumbar puncture should be performed 10–20 days after dosing.
[j]For participants enrolled in any of the PET substudies, PET may be performed after the lumbar puncture and prior to CSF results are received; there is no requirement for CSF results to be available before the PET. After aliquoting the required samples, any remaining CSF fluid from consenting participants will be kept for future RCR biomarker research.
[k]CSF samples are mandatory at Wk 104; collection at Wk 152 is optional.
[l]MRI should not be performed for 3 days following a lumbar puncture. MRI scans must be performed 20 days after dose administration and results available and reviewed before the next scheduled dose. The final MRI should be performed ~4 weeks before the final follow-up visit, to allow for review before the final visit.
[m]Includes volumetric MRI outcome measures.
[n]Includes functional MRI outcome measures.
[o]Scale requires caregiver input or support.
[p]The SymptomGuide™ Facilitated GAS will be conducted at investigational sites in French- and English-speaking countries.
[q]Study drug administration should be performed only after all assessments/rating scales for the participant are completed (unless indicated otherwise). Study drug will be administered to participants as SC injection to the abdomen. Study personnel administering study drug must not be involved with any efficacy assessments or safety evaluations. Following the first four doses, participants should be observed for a minimum of 2 hours after dosing; for the remaining doses, participants should be observed for a minimum of 1 hour. On days when only safety is being assessed, participants may have the option to have the study drug administered and applicable safety assessments conducted at a prearranged location away from the site by a trained healthcare professional, if consent is obtained.

c) OLE Years 1 and 2 (participants previously on 225 mg gantenerumab: carriers)

ADA, anti-drug antibody; ADAS-Cog 13, Alzheimer's Disease Activity Scale – Cognitive Subscale 13; ADCS-ADL, Alzheimer's Disease

Assessment/ procedure	Pre-OLE	Treatment period																	Follow-up		
		OLE 1[a]	OLW 4	OLW 8	OLW 12	OLW 16	OLW 20	OLW 24	OLW 28	OLW 32	OLW 36	OLW 40	OLW 44	OLW 48	OLW 52	OLW 53	OLWs 56–100	OLW 101	OLW 104[b]	OLW 116	UV
Dose number		1	2	3	4	5	6	7	8	9	10	11	12	13	14		15–26				
Dose level (mg)		450	450	900	900	1,200	1,200	1,200	1,200	1,200	1,200	1,200	1,200	1,200	1,200		1,200				
Informed consent(s)[c]	x																				
MRI[d]			x		x			x				x			x[e]		x[f]		x[e]		x
APOE results[g]	x																				
Vital signs[h]		x	x	x	x	x	x	x	x	x	x	x	x	x	x		x		x	x	x
ECG[i]		x												x					x		x
Serum chemistry and hematology[j]		x[k]						x						x					x		x
PK plasma sample[l]			x		x		x									x		x	x	x	x
ADA sample[l]			x																x	x	x
Urine pregnancy test[m]		x	x	x	x	x	x	x	x	x	x	x	x	x	x		x		x	x	x
Lumbar puncture[n]															x				x		x
Adverse events			x	x	x	x	x	x	x	x	x	x	x	x	x		x		x	x	x
Concomitant meds			x	x	x	x	x	x	x	x	x	x	x	x	x		x		x	x	x
ADAS-Cog 13		x[k]													x				x		x
CDR		x[k]													x				x		x
ADCS-ADL		x[k]													x				x		x
MMSE		x[k]													x				x		x
C-SSRS[o]		x						x						x			x[o]		x		x
PET scans[c]	x[p]														x				x		x
Physical exam	x[q]																		x		x

Cooperative Study – Activities of Daily Living; ALT, alanine transaminase; *APOE*, apolipoprotein E allele; ARIA, amyloid-related imaging abnormalities; AST, aspartate aminotransferase; BP, blood pressure; BUN, blood urea nitrogen; CDR, Clinical Dementia Rating; CSF, cerebrospinal fluid; C-SSRS, Columbia Suicide Severity Rating Scale; ECG, electrocardiogram; HR, heart rate; ICF, informed consent form; MMSE, Mini-Mental State Examination; MRI, magnetic resonance imaging; OLD, Open Label Day; OLE, open-label extension; OLW, Open

Label Week; PET, positron emission tomography; PK, pharmacokinetic; SGOT, serum glutamic-oxaloacetic transaminase; SGPT, serum glutamate pyruvate transaminase; UV, unscheduled visit; WBC, white blood cell.

Note: The visit window is ± 7 days for all visits.

[a]First open-label gantenerumab dose administered following signature of the OLE ICF.
[b]Participants who continue OLE beyond the initial 2 years will need to complete OLE Week 104 assessments as per Supplementary Table 2g.
[c]Informed consents have to be signed prior to participants starting open label, including an ICF to the PET substudy for those participants participating in the PET substudies. Participants who complete the initial 2 years of OLE and who will not be continuing treatment extension will have their last PET scan 1 year after their last dose.
[d]MRI scans during up-titration (including the MRI scan 3 months post 1,200 mg) or following re-dosing after ARIA findings have resolved must be performed 10–20 days after dose administration. Other scheduled MRI scans must be performed within 20 days after dose administration and results made available and reviewed before the next scheduled dose. The final MRI should be performed approximately 4 weeks before the final follow-up visit, to allow for review before the final visit.
[e]Includes volumetric and functional MRI outcome measures.
[f]A safety MRI should be collected at Week 76.
[g]*APOE* results (carrier vs noncarriers) should be revealed and appropriate counseling offered to the participant.
[h]Vital signs include HR and BP. The same arm should be used for all BP measurements. HR and BP should not be measured unless 15 minutes have passed since the last blood draw. Weight will only be collected at the OLE Week 104 visit.
[i]Perform after the participant has been in a supine position for 5 minutes. ECGs for each participant should be obtained from the same machine, whenever possible, and performed prior to any blood draws, brain MRI scans, and lumbar puncture.
[j]Serum chemistry includes AST/SGOT, ALT/SGPT, alkaline phosphatase, total protein, total bilirubin, serum albumin, creatine phosphokinase, sodium, potassium, calcium, BUN, and serum creatinine (and creatinine clearance calculated by the central laboratory). At OLE Weeks 48 and 104, hemoglobin A1c, folic acid, vitamin B12, T4, free T4, and thyroid-stimulating hormone levels will also be assessed. Hematology includes hemoglobin, hematocrit, red blood cell (with morphology), WBCs, platelet, basophil, eosinophil, lymphocyte, monocyte, neutrophil, and WBC–other total counts.
[k]Serum chemistry and hematology and listed efficacy scales should be obtained prior to first dose open label if these assessments were obtained more than 6 months ago.
[l]At all visits when PK and ADA samples (including PK obtained for ARIA findings) are needed, the samples should be obtained just before administration of gantenerumab, unless at Weeks 53 and 101. Accurate recording of the time of study drug administration and PK sampling is critical.
[m]Females of childbearing potential (including those who have had a tubal ligation) must have a urine pregnancy test performed at the site prior to each dose administration. If a urine pregnancy test is positive, it must be confirmed by a serum pregnancy test at the central laboratory.
[n]Lumbar puncture at Weeks 52 and 104 are optional. Lumbar puncture must be performed in the morning (between 8:00 a.m. and 12:00 p.m.), to minimize potential diurnal variation of CSF parameters. Lumbar puncture should be performed 10–20 days after dosing.

[o]From Weeks 56–100, C-SSRS should only be obtained at Week 72. C-SSRS may be obtained at any time deemed necessary by the investigator.
[p]Participants participating in the PET substudies and whose last PET was more than 9 months ago will need a pre-OLE PET scan prior to starting the OLE.
[q]Physical and neurologic exams prior to OLE are optional.

d) OLE Years 1 and 2 (participants previously on 225 mg gantenerumab: noncarriers)

Assessment/ procedure	Pre-OLE	Treatment period																	Follow-up		
		OLD 1[a]	OLW 4	OLW 8	OLW 12	OLW 16	OLW 20	OLW 24	OLW 28	OLW 32	OLW 36	OLW 40	OLW 44	OLW 48	OLW 52	OLW 53	OLWs 56–100	OLW 101	OLW 104[b]	OLW 116	UV
Dose number		1	2	3	4	5	6	7	8	9	10	11	12	13	14		15–26				
Dose level (mg)		600	600	1,200	1,200	1,200	1,200	1,200	1,200	1,200	1,200	1,200	1,200	1,200	1,200		1,200				
Informed consent(s)[c]	x																				
MRI[d]			x			x						x			x[e]		x[f]		x[e]		x
APOE results[g]	x																				
Vital signs[h]		x	x	x	x	x	x	x	x	x	x	x	x	x	x		x		x	x	x
ECG[i]		x												x					x		x
Serum chemistry and hematology[j]		x[k]						x						x					x		x
PK plasma sample[l]			x		x											x		x	x	x	x
ADA sample[l]			x																x	x	x
Urine pregnancy test[m]		x	x	x	x	x	x	x	x	x	x	x	x	x	x		x		x	x	x
Lumbar puncture[n]															x				x		x
Adverse events			x	x	x	x	x	x	x	x	x	x	x	x	x		x		x	x	x
Concomitant meds			x	x	x	x	x	x	x	x	x	x	x	x	x		x		x	x	x
ADAS-Cog 13		x[k]													x				x		x
CDR		x[k]													x				x		x

ADCS-ADL		x[k]			x				x	x
MMSE		x[k]			x				x	x
C-SSRS [o]		x	x	x			x[o]		x	x
PET scans [c]	x[p]				x				x	x
Physical exam	x[q]								x	x

ADA, anti-drug antibody; ADAS-Cog 13, Alzheimer's Disease Activity Scale – Cognitive Subscale 13; ADCS-ADL, Alzheimer's Disease Cooperative Study – Activities of Daily Living; ALT, alanine transaminase; *APOE*, apolipoprotein E allele; ARIA, amyloid-related imaging abnormalities; AST, aspartate aminotransferase; BP, blood pressure; CDR, Clinical Dementia Rating; C-SSRS, Columbia Suicide Severity Rating Scale; ECG, electrocardiogram; HR, heart rate; ICF, informed consent form; MMSE, Mini-Mental State Examination; MRI, magnetic resonance imaging; OLD, Open Label Day; OLE, open-label extension; OLW, Open Label Week; PET, positron emission tomography; PK, pharmacokinetic; SGOT, serum glutamic-oxaloacetic transaminase; SGPT, serum glutamate pyruvate transaminase; UV, unscheduled visit; WBC, white blood cell.

Note: The visit window is ± 7 days for all visits.

[a]First open-label gantenerumab dose administered following signature of the OLE ICF.
[b]Participants who continue OLE beyond the initial 2 years will need to complete OLE Week 104 assessments as per Supplementary Table 2g.
[c]Informed consents have to be signed prior to participants starting open label, including an ICF to the PET substudy for those participants participating in the PET substudies. Participants who complete the initial 2 years of OLE and who will not be continuing treatment extension will have their last PET scan 1 year after their last dose.
[d]MRI scans during up-titration (including the MRI scan 3 months post 1,200 mg) or following re-dosing after ARIA findings have resolved must be performed 10–20 days after dose administration. Other scheduled MRI scans must be performed within 20 days after dose administration and results made available and reviewed before the next scheduled dose. The final MRI should be performed approximately 4 weeks before the final follow-up visit, to allow for review before the final visit.
[e]Includes volumetric and functional MRI outcome measures.
[f]A safety MRI should be collected at Week 76.
[g]*APOE* results (carrier vs noncarriers) should be revealed and appropriate counseling offered to the participant.
[h]Vital signs include HR and BP. The same arm should be used for all BP measurements. HR and BP should not be measured unless 15 minutes have passed since the last blood draw. Weight will only be collected at the OLE Week 104 visit.
[i]Perform after the participant has been in a supine position for 5 minutes. ECGs for each participant should be obtained from the same machine, whenever possible, and performed prior to any blood draws, brain MRI scans, and lumbar puncture.
[j]Serum chemistry includes AST/SGOT, ALT/SGPT, alkaline phosphatase, total protein, total bilirubin, serum albumin, creatine phosphokinase, sodium, potassium, calcium, BUN, and serum creatinine (and creatinine clearance calculated by the central laboratory). At OLE Weeks 48 and

104, hemoglobin A1c, folic acid, vitamin B12, T4, free T4, and thyroid-stimulating hormone levels will also be assessed. Hematology includes hemoglobin, hematocrit, red blood cell (with morphology), WBCs, platelet, basophil, eosinophil, lymphocyte, monocyte, neutrophil, and WBC–other total counts.

[k]Serum chemistry and hematology and listed efficacy scales should be obtained prior to first dose open label if these assessments were obtained more than 6 months ago.

[l]At all visits when PK and ADA samples (including PK obtained for ARIA findings) are needed, the samples should be obtained just before administration of gantenerumab, unless at Weeks 53 and 101. Accurate recording of the time of study drug administration and PK sampling is critical.

[m]Females of childbearing potential (including those who have had a tubal ligation) must have a urine pregnancy test performed at the site prior to each dose administration. If a urine pregnancy test is positive, it must be confirmed by a serum pregnancy test at the central laboratory.

[n]Lumbar puncture at Weeks 52 and 104 are optional. Lumbar puncture must be performed in the morning (between 8:00 a.m. and 12:00 p.m.), to minimize potential diurnal variation of CSF parameters. Lumbar puncture should be performed 10–20 days after dosing.

[o]From Weeks 56–100, C-SSRS should only be obtained at Week 72. C-SSRS may be obtained at any time deemed necessary by the investigator.

[p]Participants participating in the PET substudies and whose last PET was more than 9 months ago will need a pre-OLE PET scan prior to starting the OLE.

[q]Physical and neurologic exams prior to OLE are optional.

e) OLE Years 1 and 2 (participants previously on 105 mg gantenerumab or placebo: carriers)

Assessment/ procedure	Pre-OLE	Treatment period																	Follow-up		
		OLD 1[a]	OLW 4	OLW 8	OLW 12	OLW 16	OLW 20	OLW 24	OLW 28	OLW 32	OLW 36	OLW 40	OLW 44	OLW 48	OLW 52	OLW 53	OLWs 56–100	OLW 101	OLW 104[b]	OLW 116	UV
Dose number		1	2	3	4	5	6	7	8	9	10	11	12	13	14		15–26				
Dose level (mg)		225	225	450	450	900	900	1,200	1,200	1,200	1,200	1,200	1,200	1,200	1,200		1,200				
Informed consent(s)[c]	x																				
MRI[d]			x		x					x		x[e]			x[f]		x[g]		x[f]		x
APOE results[h]	x																				
Vital signs[i]		x	x	x	x	x	x	x	x	x	x	x	x	x	x		x		x	x	x
ECG[j]		x																			
Serum chemistry and hematology[k]								x						x					x		x
PK plasma sample[l]			x		x		x		x							x		x	x	x	x
ADA sample[l]			x																x	x	x
Urine pregnancy test[m]		x	x	x	x	x	x	x	x	x	x	x	x	x	x		x		x	x	x
Lumbar puncture[n]															x				x		x
Adverse events			x	x	x	x	x	x	x	x	x	x	x	x	x		x		x	x	x
Concomitant meds			x	x	x	x	x	x	x	x	x	x	x	x	x		x		x	x	x
ADAS-Cog 13			x[o]												x				x		x
CDR			x[o]												x				x		x
ADCS-ADL			x[o]												x				x		x
MMSE			x[o]												x				x		x

C-SSRS[p]		x	x	x			x[p]		x	x
PET scans[c]	x [q]				x				x	x
Physical exam[r]	x [r]									

ADA, anti-drug antibody; ADAS-Cog 13, Alzheimer's Disease Activity Scale – Cognitive Subscale 13; ADCS-ADL, Alzheimer's Disease Cooperative Study – Activities of Daily Living; ALT, alanine transaminase; *APOE*, apolipoprotein E allele; ARIA, amyloid-related imaging abnormalities; AST, aspartate aminotransferase; BP, blood pressure; BUN, blood urea nitrogen; CDR, Clinical Dementia Rating; CSF, cerebrospinal fluid; C-SSRS, Columbia Suicide Severity Rating Scale; ECG, electrocardiogram; HR, heart rate; ICF, Informed Consent Form; MMSE, Mini-Mental State Examination; MRI, magnetic resonance imaging; OLD, Open Label Day; OLE, open-label extension; OLW, Open Label Week; PET, positron emission tomography; PK, pharmacokinetic; SGOT, serum glutamic-oxaloacetic transaminase; SGPT, serum glutamate pyruvate transaminase; UV, unscheduled visit; WBC, white blood cell.

Note: The visit window is ± 7 days for all visits.

[a]First open-label gantenerumab dose administered following signature of the OLE ICF.
[b]Participants who continue OLE beyond the initial 2 years will need to complete OLE Week 104 assessments as per Supplementary Table 2g.
[c]Informed consents have to be signed prior to participants starting open label, including an ICF to the PET substudy for those participants participating in the PET substudies. Participants who complete the initial 2 years of OLE and who will not be continuing treatment extension will have their last PET scan 1 year after their last dose.
[d]MRI scans during up-titration (including the MRI scan 3 months post 1,200 mg) or following re-dosing after ARIA findings have resolved must be performed 10–20 days after dose administration. Other scheduled MRI scans must be performed within 20 days after dose administration and results made available and reviewed before the next scheduled dose. The final MRI should be performed approximately 4 weeks before the final follow-up visit, to allow for review before the final visit.
[e]Optional MRI.
[f]Includes volumetric and functional MRI outcome measures.
[g]A safety MRI should be collected at Week 76.
[h]*APOE* results (carrier vs noncarriers) should be revealed and appropriate counseling offered to the participant.
[I]Vital signs include HR and BP. The same arm should be used for all BP measurements. HR and BP should not be measured unless 15 minutes have passed since the last blood draw. Weight will only be collected at the pre-OLE and Week 104 visits.
[j]Perform after the participant has been in a supine position for 5 minutes. ECGs for each participant should be obtained from the same machine, whenever possible, and performed prior to any blood draws, brain MRI scans, and lumbar puncture.
[k]Serum chemistry includes AST/SGOT, ALT/SGPT, alkaline phosphatase, total protein, total bilirubin, serum albumin, creatine phosphokinase, sodium, potassium, calcium, BUN, and serum creatinine (and creatinine clearance calculated by the central laboratory). At Weeks 48 and 104, hemoglobin A1c, folic acid, vitamin B12, T4, free T4, and thyroid-stimulating hormone levels will also be assessed. Hematology includes

hemoglobin, hematocrit, red blood cell (with morphology), WBCs, platelet, basophil, eosinophil, lymphocyte, monocyte, neutrophil, and WBC–other total counts.

[l]At all visits when PK and ADA samples (including PK obtained for ARIA findings) are needed, the samples should be obtained just before administration of gantenerumab, unless at Weeks 53 and 101. Accurate recording of the time of study drug administration and PK sampling is critical.

[m]Females of childbearing potential (including those who have had a tubal ligation) must have a urine pregnancy test performed at the site prior to each dose administration. If a urine pregnancy test is positive, it must be confirmed by a serum pregnancy test at the central laboratory.

[n]Lumbar puncture at Weeks 52 and 104 are optional. Lumbar puncture must be performed in the morning (between 8:00 a.m. and 12:00 p.m.), to minimize potential diurnal variation of CSF parameters. Lumbar puncture should be performed 10–20 days after dosing.

[o]Serum chemistry and hematology and listed efficacy scales should be obtained prior to first dose open label if these assessments were obtained more than 6 months ago.

[p]From Weeks 56–100, C-SSRS should only be obtained at Week 72. C-SSRS may be obtained at any time deemed necessary by the investigator.

[q]Patients participating in the PET substudies and whose last PET was more than 9 months ago will need a pre-OLE PET scan prior to starting the OLE.

[r]Physical and neurologic exams prior to OLE are optional.

f) OLE Years 1 and 2 (participants previously on 105 mg gantenerumab or placebo: noncarriers)

Assessment/ procedure	Pre-OLE	Treatment period																	Follow-up		
		OLD 1[a]	OLW 4	OLW 8	OLW 12	OLW 16	OLW 20	OLW 24	OLW 28	OLW 32	OLW 36	OLW 40	OLW 44	OLW 48	OLW 52	OLW 53	OLWs 56–100	OLW 101	OLW 104[b]	OLW 116	UV
Dose number		1	2	3	4	5	6	7	8	9	10	11	12	13	14		15–26				
Dose level (mg)		300	300	600	600	1,200	1,200	1,200	1,200	1,200	1,200	1,200	1,200	1,200	1,200		1,200				
Informed consent(s)[c]	x																				
MRI[d]			x		x			x				x			x[e]		x[f]		x[e]		x
APOE results[g]	x																				x
Vital signs[h]		x	x	x	x	x	x	x	x	x	x	x	x	x	x		x		x	x	x
ECG[i]		x												x					x		x
Serum chemistry and hematology[j]		x[k]						x						x					x		x
PK plasma sample[l]			x		x		x		x							x		x	x	x	x
ADA sample[l]			x																x	x	x
Urine pregnancy test[m]		x	x	x	x	x	x	x	x	x	x	x	x	x	x		x		x	x	x
Lumbar puncture[n]															x				x		x
Adverse events			x	x	x	x	x	x	x	x	x	x	x	x	x		x		x	x	x
Concomitant meds			x	x	x	x	x	x	x	x	x	x	x	x	x		x		x	x	x
ADAS-Cog 13			x[k]												x				x		x
CDR			x[k]												x				x		x
ADCS-ADL			x[k]												x				x		x

MMSE		x[k]			x				x	x
C-SSRS[o]		x	x	x			x[o]		x	x
PET scans[c]	x[p]				x				x	x
Physical exam[q]	x[q]								x	x

ADA, anti-drug antibody; ADAS-Cog 13, Alzheimer’s Disease Activity Scale – Cognitive Subscale 13; ADCS-ADL, Alzheimer’s Disease Cooperative Study – Activities of Daily Living; ALT, alanine transaminase; *APOE*, apolipoprotein E allele; ARIA, amyloid-related imaging abnormalities; AST, aspartate aminotransferase; BP, blood pressure; BUN, blood urea nitrogen; CDR, Clinical Dementia Rating; CSF, cerebrospinal fluid; C-SSRS, Columbia Suicide Severity Rating Scale; ECG, electrocardiogram; HR, heart rate; ICF, Informed Consent Form; MMSE, Mini-Mental State Examination; MRI, magnetic resonance imaging; OLD, Open Label Day; OLE, open-label extension; OLW, Open Label Week; PET, positron emission tomography; PK, pharmacokinetic; SGOT, serum glutamic-oxaloacetic transaminase; SGPT, serum glutamate pyruvate transaminase; UV, unscheduled visit; WBC, white blood cell.

Note: The visit window is ± 7 days for all visits.

[a]First open-label gantenerumab dose administered following signature of the OLE ICF.
[b]Participants who continue OLE beyond the initial 2 years will need to complete OLE Week 104 assessments as per Supplementary Table 2g.
[c]Informed consents have to be signed prior to participants starting open label, including an ICF to the PET substudy for those participants participating in the PET substudies. Patients who complete the initial 2 years of OLE and who will not be continuing treatment extension will have their last PET scan 1 year after their last dose.
[d]MRI scans during up-titration (including the MRI scan 3 months post 1,200 mg) or following re-dosing after ARIA findings have resolved must be performed 10–20 days after dose administration. Other scheduled MRI scans must be performed within 20 days after dose administration and results made available and reviewed before the next scheduled dose. The final MRI should be performed approximately 4 weeks before the final follow-up visit, to allow for review before the final visit.
[e]Includes volumetric and functional MRI outcome measures.
[f]A safety MRI should be collected at Week 76.
[g]*APOE* results (carrier vs noncarriers) should be revealed and appropriate counseling offered to the participant.
[h]Vital signs include HR and BP. The same arm should be used for all BP measurements. HR and BP should not be measured unless 15 minutes have passed since the last blood draw. Weight will only be collected at the pre-OLE and Week 104 visits.
[i]Perform after the participant has been in a supine position for 5 minutes. ECGs for each participant should be obtained from the same machine, whenever possible, and performed prior to any blood draws, brain MRI scans, and lumbar puncture.
[j]Serum chemistry includes AST/SGOT, ALT/SGPT, alkaline phosphatase, total protein, total bilirubin, serum albumin, creatine phosphokinase, sodium, potassium, calcium, BUN, and serum creatinine (and creatinine clearance calculated by the central laboratory). At Weeks 48 and 104, hemoglobin A1c, folic acid, vitamin B12, T4, free T4, and thyroid-stimulating hormone levels will also be assessed. Hematology includes

hemoglobin, hematocrit, red blood cell (with morphology), WBCs, platelet, basophil, eosinophil, lymphocyte, monocyte, neutrophil, and WBC–other total counts.
[k]Serum chemistry and hematology and listed efficacy scales should be obtained prior to first dose open label if these assessments were obtained more than 6 months ago.
[l]At all visits when PK and ADA samples (including PK obtained for ARIA findings) are needed, the samples should be obtained just before administration of gantenerumab, unless at Weeks 53 and 101. Accurate recording of the time of study drug administration and PK sampling is critical.
[m]Females of childbearing potential (including those who have had a tubal ligation) must have a urine pregnancy test performed at the site prior to each dose administration. If a urine pregnancy test is positive, it must be confirmed by a serum pregnancy test at the central laboratory.
[n]Lumbar puncture at Weeks 52 and 104 are optional. Lumbar puncture must be performed in the morning (between 8:00 a.m. and 12:00 p.m.), to minimize potential diurnal variation of CSF parameters. Lumbar puncture should be performed 10-20 days after dosing.
[o]From Weeks 56–100, C-SSRS should only be obtained at Week 72. C-SSRS may be obtained at any time deemed necessary by the investigator.
[p]Participants participating in the PET substudies and whose last PET was more than 9 months ago will need a pre-OLE PET scan prior to starting the OLE.
[q]Physical and neurologic exams prior to OLE are optional.

g) OLE Years 1 and 2 (all participants)

Weeks (± 7 days)		Additional years					Follow-up		UV
		104	+4–20	+24	+28–48	+52	F1[a]	F2[a] (participants not enrolling in Open RoAD)	
Informed consent[b]	x								
Dose every 4 weeks		x	x	x	x	x	x		x
MRI[c]		x[d]		x		x	x		
Vital signs[e]		x	x	x	x	x	x	x	x
ECG[f]		x				x	x		x
Serum chemistry and hematology[g]		x				x	x		x
PK plasma sample[h]		x				x	x	x	x
Urine pregnancy test[i]		x	x	x	x	x	x	x	x
Adverse events		x	x	x	x	x	x	x	x
Concomitant meds		x	x	x	x	x	x	x	x
ADAS-Cog 13		x							
CDR		x							
ADCS-ADL		x							
MMSE		x		x		x	x		x
C-SSRS [j]		x		x		x	x		x
PET scans		x				x[k]			
Physical examination		x				x	x		x
Lumbar puncture		x[l]							

ADA, anti-drug antibody; ADAS-Cog 13, Alzheimer’s Disease Activity Scale – Cognitive Subscale 13; ADCS-ADL, Alzheimer’s Disease Cooperative Study – Activities of Daily Living; ALT, alanine transaminase; ARIA, amyloid-related imaging abnormalities; AST, aspartate aminotransferase; BP, blood pressure; BUN, blood urea nitrogen; CDR, Clinical Dementia Rating; CSF, cerebrospinal fluid; C-SSRS, Columbia Suicide Severity Rating Scale; ECG, electrocardiogram; F1, Follow-Up 1; F2, Follow-Up 2 (+16 weeks after last dose); HR, heart rate; ICF, Informed Consent Form; MMSE, Mini-Mental State Examination; MRI, magnetic resonance imaging; PET, positron emission tomography; OLE, open-label extension; PK, pharmacokinetic; SGOT, serum glutamic-oxaloacetic transaminase; SGPT, serum glutamate pyruvate transaminase; UV, unscheduled visit; WBC, white blood cell.

Note: The visit window is ± 7 days for all visits. However, the minimum time between doses is 21 days, and the target day for each visit is timed with respect to baseline, not the prior visit.

[a]Follow-up visits should be obtained 4 and 16 weeks after last dose. The follow-up visit at 16 weeks is not required for participants who enroll in Open RoAD (open-label rollover study).
[b]Participants who continue OLE beyond the initial 2 years to the treatment extension should sign an ICF prior to Week 104.
[c]MRI scans during up-titration (including the MRI scan 3 months post 1,200 mg) or following re-dosing after ARIA findings have resolved must be performed 10–20 days after dose administration. Other scheduled MRI scans must be performed within 20 days after dose administration and results made available and reviewed before the next scheduled dose. The final MRI should be performed approximately 4 weeks before the final follow-up visit, to allow for review before the final visit.
[d]Includes volumetric and functional MRI outcome measures.
[e]Vital signs include HR and BP. The same arm should be used for all BP measurements. HR and BP should not be measured unless 15 minutes have passed since the last blood draw. Weight will be collected yearly at the time of physical exam.
[f]Perform after the participant has been in a supine position for 5 minutes. ECGs for each participant should be obtained from the same machine, whenever possible, and performed prior to any blood draws, brain MRI scans, and lumbar puncture.
[g]Serum chemistry includes AST/SGOT, ALT/SGPT, alkaline phosphatase, total protein, total bilirubin, serum albumin, creatine phosphokinase, sodium, potassium, calcium, BUN, and serum creatinine (and creatinine clearance calculated by the central laboratory). At OLE Weeks 48 and 104, hemoglobin A1c, folic acid, vitamin B12, T4, free T4, and thyroid-stimulating hormone levels will also be assessed. Hematology includes hemoglobin, hematocrit, red blood cell (with morphology), WBCs, platelet, basophil, eosinophil, lymphocyte, monocyte, neutrophil, and WBC–other total counts.
[h]At all visits where PK and ADA samples (including PK obtained for ARIA findings) are needed, the samples should be obtained just before administration of gantenerumab. Accurate recording of the time of study drug administration and PK sampling is critical.
[i]Females of childbearing potential (including those who have had a tubal ligation) must have a urine pregnancy test performed at the site prior to each dose administration. If a urine pregnancy test is positive, it must be confirmed by a serum pregnancy test at the central laboratory.
[j]C-SSRS should be obtained every 6 months or at any time deemed necessary by the investigator.
[k]Patients participating in the PET substudy will have a final PET scan at OLE Week 156.
[l]Lumbar puncture at Week 104 is optional. Lumbar puncture must be performed in the morning (between 8:00 a.m. and 12:00 p.m.), to minimize potential diurnal variation of CSF parameters. Lumbar puncture should be performed 10–20 days after dosing.

Supplementary Table 3

AEs occurring in ≥5% of participants in the OLE, by class and previous randomization

	Participants previously on placebo (*n* = 117)	Participants previously on gantenerumab (*n* = 108)	Total (*N* = 225)
Total number of participants with at least one AE, *n* (%)	94 (80.3)	93 (86.1)	197 (83.1)
Overall total number of events	556	598	1154
Injection site reaction, *n* (%)	48 (41.0)	44 (40.7)	92 (40.9)
ARIA-E, *n* (%)	31 (26.5)	32 (29.6)	63 (28.0)
ARIA-H, *n* (%)	19 (16.2)	24 (22.2)	43 (19.1)
Fall, *n* (%)	16 (13.7)	25 (23.1)	41 (18.2)
Headache, *n* (%)	15 (12.8)	12 (11.1)	27 (12.0)
Nasopharyngitis, *n* (%)	13 (11.1)	11 (10.2)	24 (10.7)
Urinary tract infection, *n* (%)	12 (10.3)	9 (8.3)	21 (9.3)
Constipation, *n* (%)	6 (5.1)	13 (12.0)	19 (8.4)
Diarrhea, *n* (%)	11 (9.4)	8 (7.4)	19 (8.4)
Dizziness, *n* (%)	13 (11.1)	6 (5.6)	19 (8.4)
Agitation, *n* (%)	9 (7.7)	8 (7.4)	17 (7.6)
Arthralgia, *n* (%)	8 (6.8)	7 (6.5)	15 (6.7)
Influenza, *n* (%)	4 (3.4)	10 (9.3)	14 (6.2)
Back pain, *n* (%)	8 (6.8)	5 (4.6)	13 (5.8)
Contusion, *n* (%)	5 (4.3)	8 (7.4)	13 (5.8)
Upper respiratory tract infection, *n* (%)	10 (8.5)	3 (2.8)	13 (5.8)
Insomnia, *n* (%)	7 (6.0)	5 (4.6)	12 (5.3)
Vomiting, *n* (%)	6 (5.1)	6 (5.6)	12 (5.3)
Anxiety, *n* (%)	5 (4.3)	6 (5.6)	11 (4.9)
Bronchitis, *n* (%)	6 (5.1)	5 (4.6)	11 (4.9)
Syncope, *n* (%)	5 (4.3)	6 (5.6)	11 (4.9)
Depression, *n* (%)	8 (6.8)	2 (1.9)	10 (4.4)
Edema peripheral, *n* (%)	1 (0.9)	8 (7.4)	9 (4.0)
Pyrexia, *n* (%)	2 (1.7)	6 (5.6)	8 (3.6)
Neck pain, *n* (%)	6 (5.1)	1 (0.9)	7 (3.1)

AE, adverse event; ARIA-E, amyloid-related imaging abnormalities-edema; ARIA-H, amyloid-related imaging abnormalities-hemorrhage; OLE, open-label extension.

Supplementary Table 4

ARIA-E events in the OLE by dosing step, titration regimen, and *APOE* ε4 status (safety-evaluable population with post-baseline MRI in the OLE)

Dose (mg)	I (*N* = 58)		II (*N* = 33)		III (*N* = 83)			IV (*N* = 45)		Total (*N* = 219)		
	Carrier (*n* = 58)	Total (*N* = 58)	Noncarrier (*n* = 33)	Total (*N* = 33)	Carrier (*n* = 80)	Noncarrier (*n* = 3)	Total (*N* = 83)	Noncarrier (*n* = 45)	Total (*N* = 45)	Carrier (*n* = 138)	Noncarrier (*n* = 81)	Total (*N* = 219)
**225**												
**Total no. of participants:**												
**n**	1	1	0	0	79	3	82	0	0	80	3	83
**With first ARIA-E event**	0	0	0	0	1 (1.3)	0	1 (1.2)	0	0	1 (1.3)	0	1 (1.2)
**With ARIA-E event**	0	0	0	0	2 (2.5)	1 (33.3)	3 (3.7)	0	0	2 (2.5)	1 (33.3)	3 (3.6)
**With ARIA-E event with BGTS ≤4**	0	0	0	0	2 (2.5)	0	2 (2.4)	0	0	2 (2.5)	0	2 (2.4)
**With ARIA-E leading to no up-titration**	0	0	0	0	0	0	0	0	0	0	0	0
**With ARIA-E leading to dose interruption**	0	0	0	0	1 (1.3)	0	1 (1.2)	0	0	1 (1.3)	0	1 (1.2)
**Total no. of ARIA-E events**	0	0	0	0	1	0	1	0	0	1	0	1
**300**												
**Total no. of participants:**												
**n**	0	0	2	2	1	0	1	45	45	1	47	48
**With first ARIA-E event**	0	0	0	0	0	0	0	1 (2.2)	1 (2.2)	0	1 (2.1)	1 (2.1)
**With ARIA-E event**	0	0	1 (50.0)	1 (50.0)	0	0	0	1 (2.2)	1 (2.2)	0	2 (4.3)	2 (4.2)
**With ARIA-E event with BGTS ≤4**	0	0	0	0	0	0	0	0	0	0	0	0
**With ARIA-E leading to no up-titration**	0	0	1 (50.0)	1 (50.0)	0	0	0	0	0	0	1 (2.1)	1 (2.1)
**With ARIA-E leading to dose interruption**	0	0	0	0	0	0	0	0	0	0	0	0
**Total no. of ARIA-E events**	0	0	0	0	0	0	0	1	1	0	1	1
**450**												
**Total no. of participants:**												
**n**	58	58	1	1	76	3	79	0	0	134	4	138
**With first ARIA-E event**	1 (1.7)	1 (1.7)	0	0	15 (19.7)	0	15 (19.0)	0	0	16 (11.9)	0	16 (11.6)
**With ARIA-E event**	2 (3.4)	2 (3.4)	0	0	15 (19.7)	1 (33.3)	16 (20.3)	0	0	17 (12.7)	1 (25.0)	18 (13.0)
**With ARIA-E event with BGTS ≤4**	2 (3.4)	2 (3.4)	0	0	13 (17.1)	0	13 (16.5)	0	0	15 (11.2)	0	15 (10.9)
**With ARIA-E leading to no up-titration**	1 (1.7)	1 (1.7)	0	0	9 (11.8)	0	9 (11.4)	0	0	10 (7.5)	0	10 (7.2)
**With ARIA-E leading to dose interruption**	2 (3.4)	2 (3.4)	0	0	13 (17.1)	0	13 (16.5)	0	0	15 (11.2)	0	15 (10.9)
**Total no. of ARIA-E events**	2	2	0	0	17	0	17	0	0	19	0	19
**600**												
**Total no. of participants:**												
**n**	0	0	32	32	0	0	0	43	43	0	75	75
**With first ARIA-E event**	0	0	0	0	0	0	0	4 (9.3)	4 (9.3)	0	4 (5.3)	4 (5.3)
**With ARIA-E event**	0	0	0	0	0	0	0	5 (11.6)	5 (11.6)	0	5 (6.7)	5 (6.7)
**With ARIA-E event with BGTS ≤4**	0	0	0	0	0	0	0	5 (11.6)	5 (11.6)	0	5 (6.7)	5 (6.7)
**With ARIA-E leading to no up-titration**	0	0	0	0	0	0	0	1 (2.3)	1(2.3)	0	1 (1.3)	1 (1.3)
**With ARIA-E leading to dose interruption**	0	0	0	0	0	0	0	5 (11.6)	5 (11.6)	0	5 (6.7)	5 (6.7)
**Total no. of ARIA-E events**	0	0	0	0	0	0	0	5	5	0	5	5
**900**												
**Total no. of participants:**												
**n**	55	55	2	2	65	3	68	0	0	120	5	125
**With first ARIA-E event**	6 (10.9)	6 (10.9)	0	0	6 (9.2)	0	6 (8.8)	0	0	12 (10.0)	0	12 (9.6)

**With ARIA-E event**	7 (12.7)	7 (12.7)	0	0	11 (16.9)	1 (33.3)	12 (17.6)	0	0	18 (15.0)	1 (20.0)	19 (15.2)
**With ARIA-E event with BGTS ≤4**	3 (5.5)	3 (5.5)	0	0	9 (13.8)	1 (33.3)	10 (14.7)	0	0	12 (10.0)	1 (20.0)	13 (10.4)
**With ARIA-E leading to no up-titration**	2 (3.6)	2 (3.6)	0	0	5 (7.7)	0	5 (7.4)	0	0	7 (5.8)	0	7 (5.6)
**With ARIA-E leading to dose interruption**	3 (5.5)	3 (5.5)	0	0	8 (12.3)	1 (33.3)	9 (13.2)	0	0	11 (9.2)	1 (20.0)	12 (9.6)
**Total no. of ARIA-E events**	8	8	0	0	8	0	8	0	0	16	0	16
**1200**												
**Total no. of participants:**												
**n**	54	54	30	30	58	2	60	39	39	112	71	183
**With first ARIA-E event**	15 (27.8)	15 (27.8)	9 (30.0)	9 (30.0)	7 (12.1)	0	7 (11.7)	5 (12.8)	5 (12.8)	22 (19.6)	14 (19.7)	36 (19.7)
**With ARIA-E event**	18 (33.8)	18 (33.8)	9 (30.0)	9 (30.0)	11 (19.0)	0	11 (18.3)	5 (12.8)	5 (12.8)	29 (25.9)	14 (19.7)	43 (23.5)
**With ARIA-E event with BGTS ≤4**	12 (22.2)	12 (22.2)	8 (26.7)	8 (26.7)	9 (15.5)	0	9 (15.0)	5 (12.8)	5 (12.8)	21 (18.8)	13 (18.3)	34 (18.6)
**With ARIA-E leading to no up-titration**	0	0	0	0	0	0	0	0	0	0	0	0
**With ARIA-E leading to dose interruption**	12 (22.2)	12 (22.2)	8 (26.7)	8 (26.7)	9 (15.5)	0	9 (15.0)	5 (12.8)	5 (12.8)	21 (18.8)	13 (18.3)	34 (18.6)
**Total no. of ARIA-E events**	19	19	10	10	11	0	11	5	5	30	15	45
**Total**												
**Total no. of participants:**												
**n**	58	58	33	33	80	3	83	45	45	138	81	219
**With first ARIA-E event**	22 (37.9)	22 (37.9)	9 (27.3)	9 (27.3)	29 (36.3)	0	29 (34.9)	10 (22.2)	10 (22.2)	51 (37.0)	19 (23.5)	70 (32.0)
**With ARIA-E event with BGTS ≤4**	16 (27.6)	16 (27.6)	8 (24.2)	8 (24.2)	27 (33.8)	1 (33.3)	28 (33.7)	10 (22.2)	10 (22.2)	43 (31.2)	19 (23.5)	62 (28.3)
**With ARIA-E leading to no up-titration**	3 (5.2)	3 (5.2)	1 (3.0)	1 (3.0)	14 (17.5)	0	14 (16.9)	1 (2.2)	1 (2.2)	17 (12.3)	2 (2.5)	19 (8.7)
**With ARIA-E leading to dose interruption**	16 (27.6)	16 (27.6)	8 (24.2)	8 (24.2)	27 (33.8)	1 (33.3)	28 (33.7)	10 (22.2)	10 (22.2)	43 (31.2)	19 (23.5)	62 (28.3)
**Total no. of ARIA-E**	29	29	10	10	37	0	37	11	11	66	21	87

All values are *n* (%) unless otherwise stated.

APOE ε4, apolipoprotein E ε4 allele; ARIA-E, amyloid-related imaging abnormalities – edema; MRI, magnetic resonance imaging; OLE, open-label extension.

Supplementary Table 5

AEs associated with ARIA-E MRI findings and retrieved using a post hoc programmatic approach (safety-evaluable participants with post-baseline MRI in OLE)

	Total (N = 219)
Total number of participants with at least one AE reported as symptoms of ARIA-E	22 (10.0)
Overall total number of symptoms associated with ARIA-E	33
Headache	4 (1.8)
Dizziness	3 (1.4)
Syncope	3 (1.4)
Cognitive disorder	2 (0.9)
Dysesthesia	1 (0.5)
Dyskinesia	1 (0.5)
Epilepsy	1 (0.5)
Generalized tonic-clonic seizure	1 (0.5)
Hemianopia	1 (0.5)
Hemiplegia	1 (0.5)
Hydrocephalus	1 (0.5)
Loss of consciousness	1 (0.5)
Myoclonus	1 (0.5)
Patient elopement	1 (0.5)
Presyncope	1 (0.5)
Psychomotor hyperactivity	1 (0.5)
Subarachnoid hemorrhage	1 (0.5)
Tongue paralysis	1 (0.5)
White matter lesion	1 (0.5)

All values are *n* (%) unless otherwise stated.
AE, adverse event; ARIA-E, amyloid-related imaging abnormalities-edema; MRI, magnetic resonance imaging; OLE, open-label extension.

Supplementary Table 6

Summary of efficacy results and change from baseline values to Week 104 using MMRM analysis in double-blind part

Efficacy endpoint	Placebo (*n*)	Gantenerumab (*n*)	Mean difference (SE)	95% CI	*p*
ADAS-Cog 13	30	16	–3.71 (1.93)	–7.54, 0.12	0.058
ADCS-ADL total score	28	15	3.84 (2.58)	–1.29, 8.97	0.267
CDR-SB	29	17	–0.25 (0.60)	–1.45, 0.94	0.672
MMSE	29	16	0.55 (0.91)	–1.27, 2.37	0.548

ADAS-Cog 13, Alzheimer’s Disease Assessment Scale – Cognitive Subscale 13; ADCS-ADL, Alzheimer's Disease Cooperative Study – Activities of Daily Living; CDR-SB, Clinical Dementia Rating – Sum of Boxes; CI, confidence interval; MMRM, Mixed Model Repeated Measures; MMSE, Mini-Mental State Examination; OLE, open-label extension; SE, standard error.

Supplementary Table 7

Summary of efficacy outcomes and change from OLE baseline at Week 104 by previous randomization (treated OLE participants)

	Participants previously on placebo (*n* = 117)		Participants previously on gantenerumab (*n* = 108)		Total (*N* = 225)	
Efficacy endpoint	Value at visit	Change from baseline	Value at visit	Change from baseline	Value at visit	Change from baseline
CDR-SB						
n	60	60	54	54	114	114
Mean (SD)	8.33 (3.67)	3.37 (2.80)	9.20 (4.49)	4.19 (3.71)	8.75 (4.08)	3.76 (3.27)
Median	8.00	3.00	8.50	3.25	8.50	3.00
Q1–Q3	5.00, 11.00	1.50, 6.00	6.00, 12.00	1.00, 6.00	5.50, 11.00	1.00, 6.00
Min–Max	2.0, 16.0	–2.0, 9.5	0.0, 18.0	–2.0, 13.5	0.0, 18.0	–2.0, 13.5
CDR-GS						
n	60	60	54	54	114	114
Mean (SD)	1.39 (0.60)	0.57 (0.55)	1.56 (0.84)	0.69 (0.78)	1.47 (0.73)	0.62 (0.67)
Median	1.00	0.50	1.00	0.50	1.00	0.50
Q1–Q3	1.00, 2.00	0.00, 1.00	1.00, 2.00	0.00, 1.00	1.00, 2.00	0.00, 1.00
Min–Max	0.5, 3.0	–0.5, 2.0	0.0, 3.0	–1.0, 2.5	0.0, 3.0	–1.0, 2.5
MMSE						
n	60	60	51	51	111	111
Mean (SD)	16.65 (6.59)	–4.53 (4.07)	15.27 (6.86)	–5.96 (5.30)	16.02 (6.72)	–5.19 (4.70)
Median	17.00	–4.00	16.00	–4.00	17.00	–4.00
Q1–Q3	11.50, 22.00	–7.00, –2.00	11.00, 21.00	–8.00, –2.00	11.00, 22.00	–8.00, –2.00
Min–Max	3.0, 28.0	–16.0, 5.0	0.0, 30.0	–23.0, –2.0	0.0, 30.0	–23.0, –5.0
ADAS-Cog 13						
n	59	59	52	52	111	111
Mean (SD)	43.80 (15.76)	11.39 (12.52)	45.63 (18.86)	13.38 (15.41)	44.66 (17.23)	12.32 (13.92)
Median	42.00	9.00	40.50	8.50	41.00	9.00
Q1–Q3	32.00, 56.00	2.00, 16.00	33.50, 57.00	3.00, 20.50	33.00, 56.00	3.00, 20.00
Min–Max	20.0, 80.0	–14.0, 42.0	9.0, 85.0	–28.0, 56.0	9.0, 85.0	–28.0, 56.0
ADCS-ADL						
n	59	59	50	50	109	109
Mean (SD)	49.76 (17.04)	–12.25 (12.48)	47.22 (19.20)	–14.78 (16.70)	48.60 (18.02)	–13.41 (14.55)
Median	54.00	–11.00	52.00	–10.00	53.00	–10.00
Q1–Q3	36.00, 63.00	–19.00, –3.00	37.00, 61.00	–25.00, –3.00	37.00, 63.00	–21.00, –3.00
Min–Max	12.0, 76.0	–48.0, 6.0	6.0, 77.0	–63.0, –8.0	6.0, 77.0	–63.0, 8.0

ADAS-Cog 13, Alzheimer's Disease Assessment Scale – Cognitive Subscale 13; ADCS-ADL, Alzheimer's Disease Cooperative Study – Activities of Daily Living; CDR-GS, Clinical Dementia Rating – Global Score; CDR-SB, Clinical Dementia Rating – Sum of Boxes; MMSE, Mini-Mental State Examination; OLE, open-label extension; SD, standard deviation.

Supplementary Table 8

MMSE total scores and change from OLE baseline by visit past Week 104 by previous randomization (safety-evaluable population)

	Participants previously on placebo (*n* = 117)		Participants previously on gantenerumab (*n* = 108)		Total (*N* = 225)	
Visit	Value at visit	Change from baseline	Value at visit	Change from baseline	Value at visit	Change from baseline
Week 128						
n	60	60	44	44	104	104
Mean (SD)	15.10 (6.42)	–6.12 (4.19)	15.43 (6.50)	–6.18 (4.98)	15.24 (6.42)	–6.14 (4.52)
Median	15.50	–6.00	15.50	–6.00	15.50	–6.00
Q1–Q3	10.00, 21.00	–8.00, –3.50	10.50, –20.00	–9.00, –2.00	10.00, 20.50	–8.00, –3.00
Min–Max	0.0, 27.0	–20.0, 3.0	1.0, 30.0	–19.0, 2.0	0.0, 30.0	–20.0, 3.0
Week 156						
n	49	49	36	36	85	85
Mean (SD)	14.37 (7.04)	–6.73 (5.44)	14.39 (6.07)	–6.94 (5.68)	14.38 (6.86)	–6.82 (4.52)
Median	14.00	–7.00	16.00	–6.00	15.00	–7.00
Q1–Q3	8.00, 21.00	–9.00, –4.00	9.50, 20.00	–11.00, –2.00	8.00, 20.00	–10.00, –4.00
Min–Max	0.0, 29.0	–19.0, 8.0	1.0, 24.0	–21.0, 1.0	0.0, 29.0	–21.0, 8.0
Week 180						
n	40	40	28	28	68	68
Mean (SD)	13.95 (7.90)	–7.55 (5.43)	13.18 (6.99)	–8.07 (5.64)	13.63 (7.49)	–7.76 (5.49)
Median	14.50	–7.00	15.00	–7.50	15.00	–7.00
Q1–Q3	7.50, 21.00	–10.50, –3.00	8.50, 18.50	–13.00, –3.00	8.00, 20.00	–11.00, –3.00
Min–Max	0.0, 26.0	–21.0, 2.0	1.0, 24.0	–19.0, –1.0	0.0, 26.0	–21.0, 2.0
Week 208						
n	25	25	22	22	47	47
Mean (SD)	12.04 (7.35)	–9.48 (3.96)	14.32 (6.13)	–7.59 (5.81)	13.11 (6.83)	–8.60 (4.95)
Median	12.00	–9.00	–9.00	–6.00	15.00	–9.00
Q1–Q3	4.00, 18.00	–11.00, –7.00	10.00, 18.00	–12.00, –3.00	7.00, 18.00	–11.00, –5.00
Min–Max	0.0, 22.0	–21.0, –3.0	3.0, 24.0	–20.0, 1.0	0.0, 24.0	–21.0, 1.0
Week 232						
n	14	14	14	14	28	28
Mean (SD)	12.93 (7.14)	–9.43 (5.24)	12.00 (7.32)	–10.43 (6.72)	12.46 (7.11)	–9.93 (5.94)
Median	15.50	–8.50	13.50	–9.00	14.00	–8.50
Q1–Q3	6.00, 18.00	–11.00, –7.00	6.00, 18.00	–15.00, –5.00	6.00, 18.00	–13.00, –6.00
Min–Max	0.0, 21.0	–21.0, –3.0	0.0, 24.0	–22.0, –1.0	0.0, 24.0	–22.0, –1.0

MMSE, Mini-Mental State Exam; MRI, magnetic resonance imaging; OLE, open-label extension.

Supplementary Table 9

Summary statistics of gantenerumab plasma concentrations per scheduled visit in the OLE

Nominal time (weeks)	Dose level[b] (mg)	Number of samples	Mean (µg/mL)	SD (µg/mL)	Median (µg/mL)	Min (µg/mL)	Max (µg/mL)	CV (%)
Group 1: *APOE* ε4 carriers treated with 225 mg gantenerumab until the end of the DB part								
4	405	59	10.2	4.39	9.82	0.662	27.5	42.9
12	900	50	20.7	9.51	18.8	4.98	51.1	46
20	1,200	44	27.8	11.9	27.6	4.89	69.1	43
Group 2: *APOE* ε4 noncarriers treated with 225 mg gantenerumab until the end of the DB part								
4	600	31	12.6	6.69	11.4	2.3	35.4	52.9
12	1,200	27	33.4	16.3	27.9	12.9	72.9	48.9
Group 3: *APOE* ε4 carriers treated with placebo or 105 mg gantenerumab until the end of the DB part								
4	225	81	4.17	1.92	3.74	0.606	11.6	46.1
12	450	71	12.4	5.12	12.2	1.8	29	41.2
20	900	58	22.1	11.5	19.5	3.83	56.7	51.9
28	1,200	40	30.7	11.7	31.3	4.39	58.3	38.1
Group 4: *APOE* ε4 noncarriers treated with placebo or 105 mg gantenerumab until the end of the DB part								
4	225	42	4.3	1.77	4.34	1.06	7.87	41.1
12	600	43	13.9	6.69	12.8	3.52	34.5	48.3
20	1,200	34	29.5	10.5	27.1	9.81	50.3	35.6
All participants (same treatment schedule for the remainder of the study)								
53[a]	1,200	128	80.6	38.4	74.3	1.15	160	47.7
101[a]	1,200	111	89.1	33.6	89.1	7.04	181	37.7
104	1,200	141	43.5	22.4	40.4	0.287	135	51.5
116	1,200	15	3.66	2.29	3.17	0.126	8.24	62.4
156	1,200	80	45.2	22.5	41.7	8.69	117	49.9
208	1,200	48	55.8	37.9	47.7	0.028	147	67.9

[a]Samples taken approximately 1 week post dosing.

[b]Concentration measurements from participants who were not on the scheduled dose level were excluded: 216 unscheduled concentration measurements were excluded from the summary statistics.

APOE ε4, apolipoprotein E ε4 allele; CV, coefficient of variation; DB, double blind; OLE, open-label extension; SD, standard deviation.
